# Simulating Free-Roaming Cat Population Management Options in Open Demographic Environments

**DOI:** 10.1371/journal.pone.0113553

**Published:** 2014-11-26

**Authors:** Philip S. Miller, John D. Boone, Joyce R. Briggs, Dennis F. Lawler, Julie K. Levy, Felicia B. Nutter, Margaret Slater, Stephen Zawistowski

**Affiliations:** 1 Conservation Breeding Specialist Group, Species Survival Commission, International Union for Conservation of Nature, Apple Valley, Minnesota, United States of America; 2 Great Basin Bird Observatory, Reno, Nevada, United States of America; 3 Alliance for Contraception in Cats and Dogs, Portland, Oregon, United States of America; 4 Illinois State Museum, Springfield, Illinois, United States of America; 5 Maddie's Shelter Medicine Program, Department of Small Animal Clinical Sciences, University of Florida, Gainesville, Florida, United States of America; 6 Department of Infectious Disease and Global Health, Cummings School of Veterinary Medicine, Tufts University, North Grafton, Massachusetts, United States of America; 7 Shelter Research and Development, American Society for the Prevention of Cruelty to Animals, Florence, Massachusetts, United States of America; 8 American Society for the Prevention of Cruelty to Animals, New York, New York, United States of America; University of Queensland, Australia

## Abstract

Large populations of free-roaming cats (FRCs) generate ongoing concerns for welfare of both individual animals and populations, for human public health, for viability of native wildlife populations, and for local ecological damage. Managing FRC populations is a complex task, without universal agreement on best practices. Previous analyses that use simulation modeling tools to evaluate alternative management methods have focused on relative efficacy of removal (or trap-return, TR), typically involving euthanasia, and sterilization (or trap-neuter-return, TNR) in demographically isolated populations. We used a stochastic demographic simulation approach to evaluate removal, permanent sterilization, and two postulated methods of temporary contraception for FRC population management. Our models include demographic connectivity to neighboring untreated cat populations through natural dispersal in a metapopulation context across urban and rural landscapes, and also feature abandonment of owned animals. Within population type, a given implementation rate of the TR strategy results in the most rapid rate of population decline and (when populations are isolated) the highest probability of population elimination, followed in order of decreasing efficacy by equivalent rates of implementation of TNR and temporary contraception. Even low levels of demographic connectivity significantly reduce the effectiveness of any management intervention, and continued abandonment is similarly problematic. This is the first demographic simulation analysis to consider the use of temporary contraception and account for the realities of FRC dispersal and owned cat abandonment.

## Introduction

Free-roaming cats (FRCs) are distributed world-wide in populations that occupy diverse habitats, often at high densities [1]. The abundance of unowned free-roaming cats in the United States is estimated in the tens of millions [2]. While 80% of owned cats in the United States are surgically sterilized [3], most unowned cats remain reproductively active, facilitating FRC population growth where resources and habitat permit. FRC populations prompt concerns about animal welfare [4], human public health [5],[6], and threats to native wildlife from predation and disease transmission [7]–[11].

Management of FRC populations takes a variety of forms around the world. In the United States, management of unowned cats in FRC populations traditionally has relied on trap and removal (TR), where a large proportion of cats are typically euthanized, or for a smaller portion of socialized cats, adopted. Trap-neuter-return (TNR) uses surgical sterilization in combination with natural mortality of cats in their native environment to reduce population abundance [12]. The relative efficacy of TR and TNR strategies has been evaluated using various computer simulation models that differ in their underlying structures, mechanics, and assumptions [13]–[22].

Most FRC population models make simplifying assumptions about population dynamics, presumably to manage mathematical complexity in the models themselves. For example, most simulated populations are demographically isolated (i.e., an island scenario) without recruitment of new individuals through immigration or abandonment of unwanted cats by humans. In reality, unowned FRC populations typically are not isolated but would instead interact demographically with cats in surrounding landscapes. This natural connectivity may play a significant role in the long-term dynamics of a population that is subjected to management by such methods as TR and TNR. Finally, most previous modeling efforts have not explicitly incorporated density-dependent population regulatory mechanisms. Simulating one or more forms of density dependence contributes ecological realism to all such analyses and greater analytical rigor to the resulting management recommendations.

Ethical objections in some cultures to lethal control and concerns about the logistical demands and expense of large-scale surgical sterilization programs have combined to stimulate interest in the development of non-surgical contraceptive methods that can be administered in field settings [23]. Final development of new non-surgical contraceptive options could be accelerated if rigorous models suggest the potential for population-level efficacy and efficiency under realistic biological scenarios. To date, detailed quantitative analyses of potential impacts of permanent sterilization vs. temporary contraception on FRC population dynamics have not been reported.

We describe an analysis using stochastic simulation modeling to evaluate multiple management options for FRC populations in human-modified landscapes. We model TR, TNR, and two hypothesized methods for temporary contraception, and explicitly incorporate immigration, litter abandonment, and density-dependent population regulation within the modeling framework. Our goal is to provide practical guidance to the cat population management community, based on sound application of rigorous quantitative methods for data assembly and analysis.

## Methods

**Figure 1:**
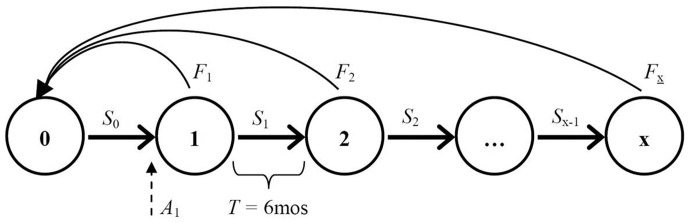
Generalized life-cycle diagram depicting free-roaming cat (FRC) population demographics used in simulation models. Numbers refer to specific age cohorts, separated in age by timestep *T* = 6 months. Parameter *S_i_* denotes age-specific 6-month survival rates, while *F_i_* denotes reproductive rates across age classes. Abandonment of owned litters, as a contribution to the focal population, is represented by quantity *A*_1_.

We developed our models using the individual-based stochastic simulation software package *Vortex*, version 9.99b [24]. Our detailed demographic models featured an explicit age/sex structure that allowed specification of birth and death rates as a function of individual identity (Figure 1). Additionally, we adopted a discrete 6-month timestep to accommodate seasonal reproduction among cats living in temperate environments (Figure S1; Table S1). Our models also featured a metapopulation approach by incorporating demographic connectivity between a focal cat population undergoing treatment and cat populations in the surrounding “neighborhood”. We further assume that FRCs display strongly polygynous breeding behaviors, with little to no social stratification among breeding males [25],[26]. Consequently, we assumed that male fecundity does not limit population growth. Because of our assumption concerning male breeding behavior, we did not evaluate reproductive management options, such as trap-vasectomy-hysterectomy-release (TVHR), that attempt to maintain normal reproductive hormone levels and social behavior [27].

All our models feature density dependence in the form of increased kitten mortality with increasing population density (Figure S2). Additionally, we impose a simple ceiling mode of density dependence by specifying a carrying capacity for each habitat selected for our study (see below). Other forms of density dependence, such as increased kitten and/or adult survival with increasing density of neutered individuals proposed by other researchers [17],[28], were not included in our models as the precise nature of this functional relationship is not clear. Finally, the suggestion that neutered cats display markedly greater longevity [17] was not incorporated into this model because of the difficulty in interpreting the data in terms of actual changes in survival rate or, alternatively, simple differences in colony residence time among neutered and intact cats.

**Figure 2:**
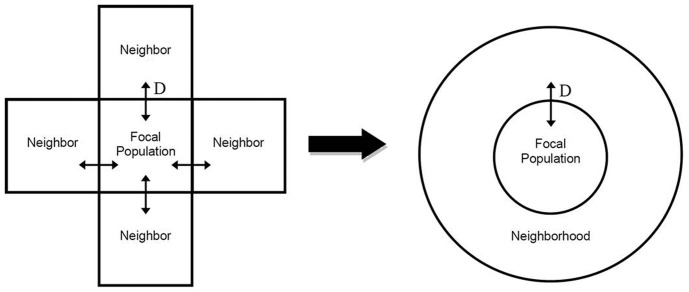
FRC metapopulation structure used in simulation models. Spatial representation (*left*) of a focal FRC population in an area surrounded by similar habitat inhabited by untreated cats, and a generalized representation (*right*) of that same metapopulation configuration used within the *Vortex* simulation environment. Dispersal rate designated by parameter *D*.

Evaluating a focal population and surrounding “neighborhood” requires considering the spatial extent in the model system. Our focal population was 50 Ha = 0.5 km^2^ in size (approximately 25 urban square blocks), while the neighborhood was four times larger in spatial extent (Figure 2). We simulated movement between these metapopulation components by specifying age/sex-specific dispersal rates (*D*). In addition, for select simulations, we added four 6-month-old kittens (2 male, 2 female) to the focal population at each timestep. This addition simulates abandonment by humans, a factor that is important in FRC population dynamics [7],[12], but is not addressed in other population dynamics models. Abandonment of older individuals certainly occurs, but we assumed this to be a comparatively rare event and therefore one not explicitly included in our analysis.

We structured our full set of analyses around three broadly categorized FRC population types defined by density as a function of resource availability, habitat availability, and extent of demographic isolation (Table S2):

Large Urban – relatively large population, dense urban setting, high resource availability through human interaction (supplemental feeding, access to garbage, abundant shelter), leading to both higher survivals and population densities. The focal population was connected to surrounding neighborhoods through dispersal, and litter abandonment was added.
Small Urban – town or village population with some resource availability, leading to similar survival rates to the Large Urban setting, but at lower total numbers. The focal population was connected to surrounding neighborhoods through dispersal, and litter abandonment was added.
Rural – smaller population, farm or small natural area, limited resource availability, lower survival across ages, and lower population densities. We assume isolation from other populations by means of a matrix of unsuitable habitat (no metapopulation structure), and litter abandonment was not added.

We then applied each of the following four management options to each of the population types:

Remove: Cats were trapped and permanently removed from the population without specifying their fate, representing TR programs.
Sterilize: Cats were trapped, permanently sterilized through castration of males and ovariohysterectomy of females, and returned to their population of origin, representing TNR programs.
Contracept-A: Cats were trapped, non-surgically contracepted, and returned to their population of origin. Each treated individual was temporarily infertile; contracepted cats returned to full reproductive activity three years after contraception, and were subject to further trapping and potential treatment. A contraceptive with this specific mode of action does not yet exist; we included this hypothesized option to conform to a previous modeling study [14] featuring this alternative.
Contracept-B: Cats were treated as in Contracept-A, but returned to reproductive activity probabilistically over a period of six months to five years following initial treatment (Figure S3), simulating laboratory results using a GnRH immunocontraceptive (GonaCon^TM^) [29],[30]. Animals reverting to reproductive activity were subject to further trapping and potential treatment.

In all four management options, specified proportions (10%–50%) of kittens (age 6 months), adults (age >6 months), or all cats, were treated each timestep. Individuals were selected at random to simulate trapping events, with no quantitative reference made in the model to capture probability. Selection of individuals continued at each time step until the desired proportion of untreated individuals were “trapped” and treated, meaning that previously treated cats could be trapped again and not subject to treatment.

We employed elasticity (proportional sensitivity) analysis [31] to identify the major demographic factors driving simulated FRC population growth. To perform this analysis, we created a simple, female-only stage-based demographic transition matrix using the simulation software package *RAMAS Metapop*, Version 5.02 [32]. This matrix described average reproductive and survival rates of kittens and adults inhabiting a Large Urban landscape and not subject to any form of population management (Table S3). Using this matrix, elasticity *E*(*x*) is calculated using the expression
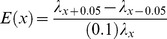
where λ*_x_*_+0.05_ is the deterministic growth rate calculated from the above matrix with parameter *x* increased by 5%, λ*_x_*_−0.05_ is the deterministic growth rate calculated with parameter *x* decreased by 5%, and λ*_x_* is the deterministic growth rate calculated from the unmodified matrix. Using this formulation, a larger elasticity value for a given demographic parameter indicates a greater level of sensitivity within the demographic model to change in that parameter.

Each scenario was run for 100 timesteps (50 years) and repeated with 1000 iterations to generate statistics on mean population behavior. Additional detailed information on model structure and input data analysis is available in the Supporting Information.

## Results

### Population growth in the absence of treatment

Without population management, simulated Large and Small Urban cat populations were capable of growing 18–20% per year (8.5–10% per 6-month timestep), given sufficient resources. This simulated growth rate is consistent with those obtained from field studies of FRC populations inhabiting similar environments [15],[33], thereby giving our models a high level of biological realism. Habitat-specific carrying capacities ultimately constrained long-term abundance. The isolated Rural population grew about 5.5% annually (2.7% per timestep).

### Elasticity analysis

Standard elasticity analysis indicated that our models were more sensitive to changes in age-specific survival rates than to age-specific reproductive rates (fecundity), with adult survival showing the greatest impact on population size (Figure S4). Specifically, elasticity for adult survival was calculated to be 0.573, while the values for both kitten survival and adult fecundity were calculated to 0.184. The impact on a FRC population's growth rate resulting from a proportional change in adult annual survival will be more than three times greater than the impact of an identical proportional change in adult female reproductive output. This is explained most effectively by high kitten mortality rates and multiple opportunities for breeding across adult lifespan.

### Treatment impact: Demographic isolation

**Figure 3:**
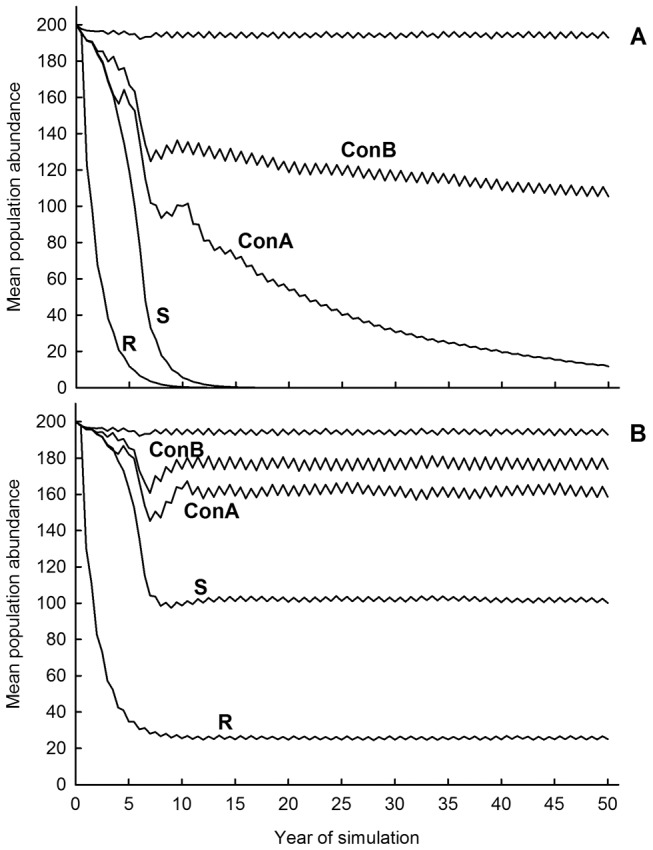
Impact of population management options on simulated FRC abundance. (A) Fifty-year mean abundance trajectories for a simulated Large Urban population subject to different population management strategies at a rate of 40% treatment of all untreated individuals each timestep. Simulations include demographic isolation. Uppermost trajectory is the baseline, no-treatment scenario. (B) Abundance trajectories as above but with demographic connectivity (dispersal, litter abandonment). R, Remove; S, Sterilize; ConA, Contracept-A; ConB, Contracept-B.

When imposing high (40%) treatment intensity on an isolated Large Urban population, the TR and TNR options led to rapid population decline, with elimination by 10–15 years (Figure 3A). Temporary hypothesized contraception options reduced population size nearly as well as did sterilization in the short-term (<5 years), but not in the longer-term (>10 years). Elimination probability under Contracept-A was just under 60% (mean time to elimination = 36 years), while elimination probability under Contracept-B was 0%.

### Treatment impact: Demographic connectivity

With demographic connectivity, TR and TNR remained the most effective options for reducing population size over the long term (Figure 3B). However, imposition of connectivity with the surrounding neighborhood made population elimination impossible. Instead, average long-term population abundance reaches an equilibrium value that is a function of the type of management employed, with the increased functional mortality imposed by TR leading to the lowest equilibrium abundance.

### Consistency of model results

**Figure 4:**
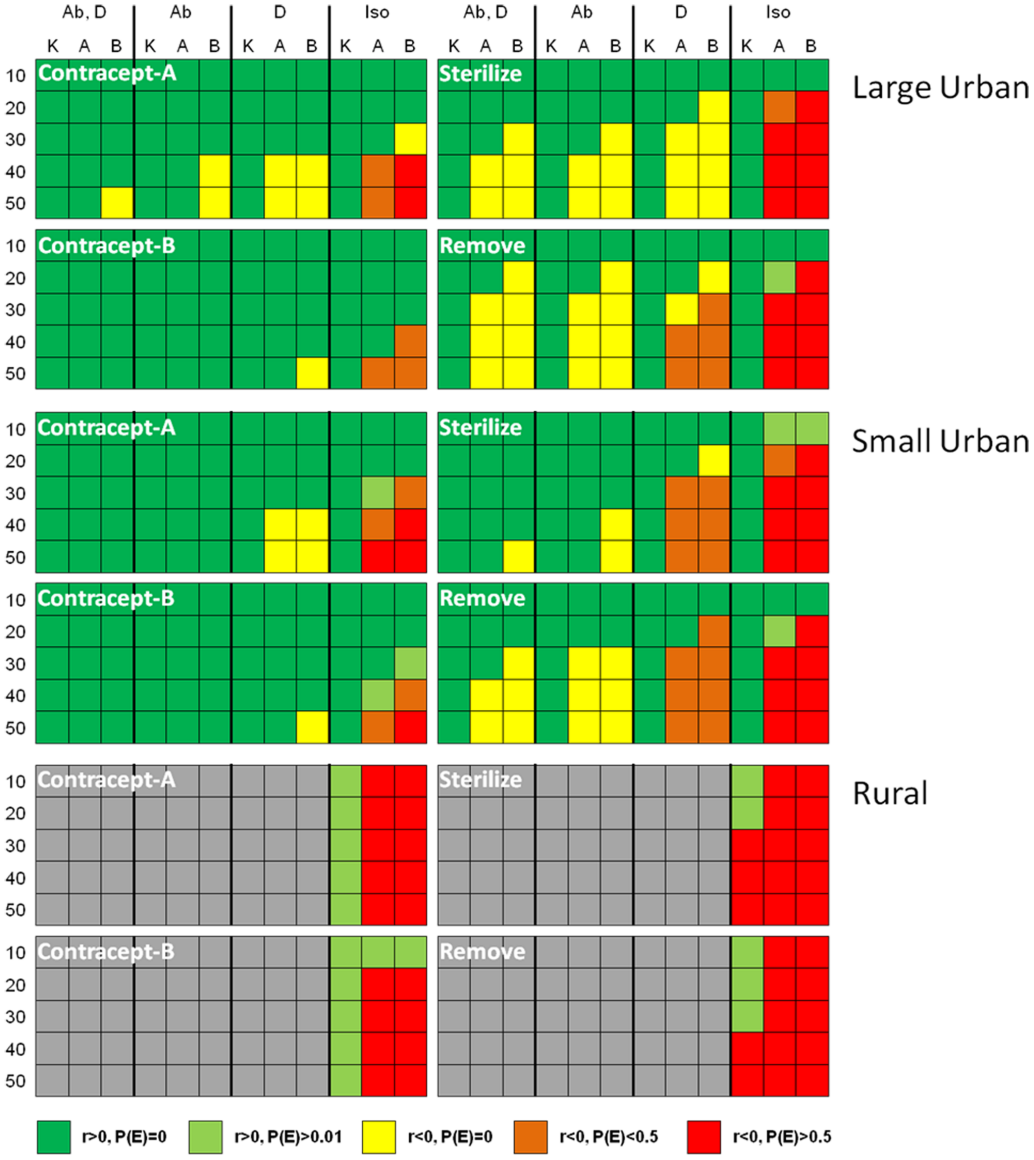
Comparative performance of simulated FRC management options across population types. Row headings define the rate of treatment of individuals, as percentage of untreated kittens (K), adults (A), or both (B) treated each 6-month timestep. Column headings identify the inclusion of specific population connectivity characteristics in a given scenario: litter abandonment (Ab), dispersal to the surrounding neighborhood population (*D*), or population isolation (Iso). Each cell is color-coded based on the combined result of a specific model scenario, defined in terms of the mean stochastic growth rate (*r*) over the 50-year timeframe of the simulation and the risk of population elimination (P(E)) within that same time period (see color key at bottom of figure). Cells shaded gray represent scenarios that were not evaluated in this analysis.

**Figure 5:**
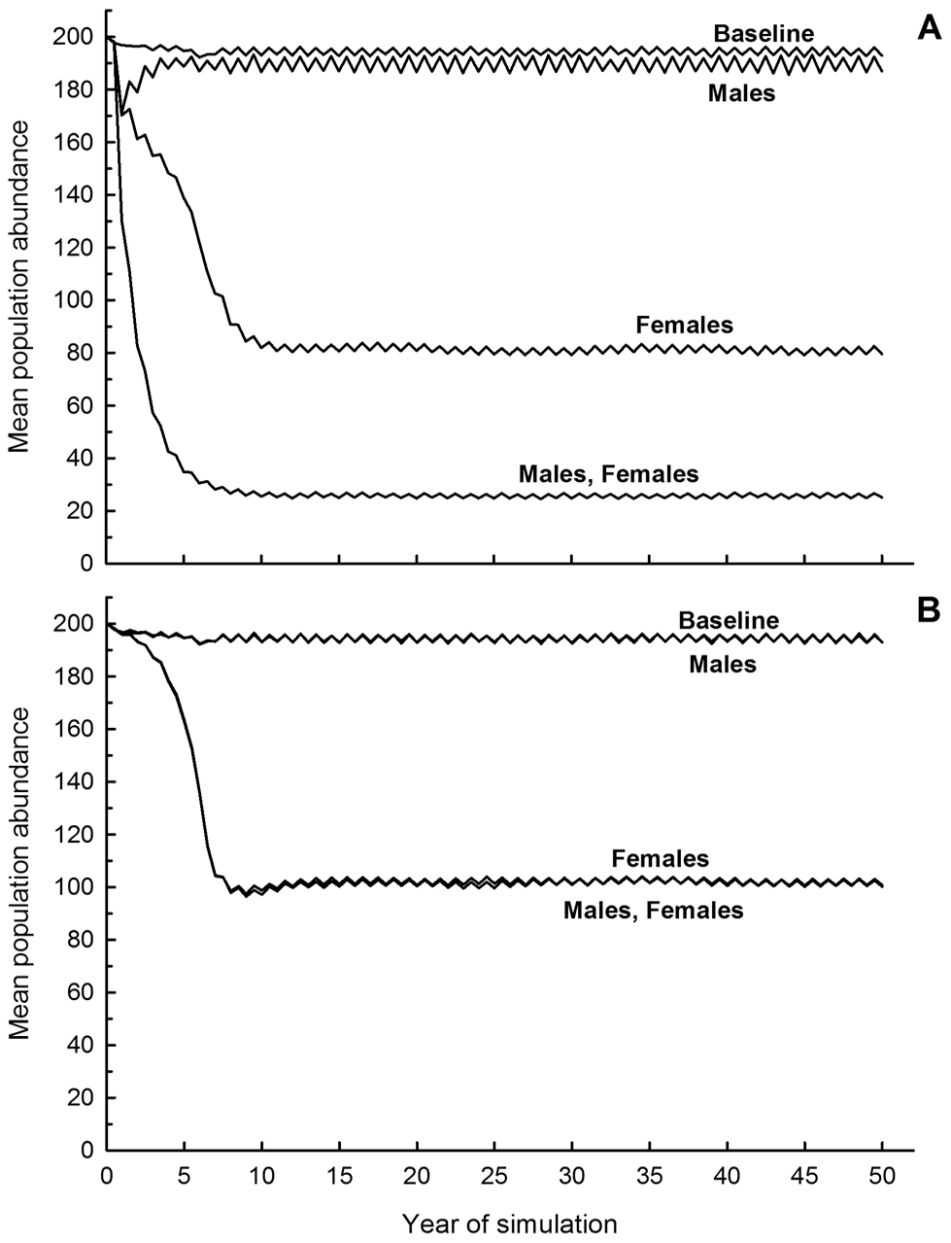
Impact of gender-specific management strategies on simulated FRC abundance. Fifty-year mean abundance trajectories for a simulated Large Urban free-roaming cat population subject to 40% Removal (A, top) or Sterilization (B, bottom) of adults each timestep. Separate models feature exclusive trapping of males or females in addition to the standard scenarios featuring indiscriminant trapping across gender. Baseline models feature no management imposed on the population.

Several results were generally consistent across the suite of 540 scenarios assessed in this study (Figure 4; Tables S4–S15):

- Within population type, TR led to more outcomes of population decline and, in isolated populations, measurable elimination probability, followed in order of decreasing effectiveness by TNR, Contracept-A, and Contracept-B.
- Treating kittens exclusively was much less effective in reducing population growth rate than treating an equivalent proportion of adults.
- Because of our assumptions about underlying male breeding patterns (i.e., little to no social hierarchy among breeding males), sterilization or contraception of males had a negligible impact on long-term population size (Figure 5).
- Even modest demographic connectivity had a profound buffering effect on efforts to reduce FRC population size.
- Under the conditions evaluated in this study, litter abandonment proved to be a more effective mechanism of demographic connectivity than was dispersal to and from the surrounding neighborhood. This is likely explained by the fact that dispersal was assumed to be a stochastic process, resulting in little to no movement of animals into the focal population by chance in some years. In contrast, the abandonment of four kittens from households within the focal population area at each timestep was considered to be a deterministic process, and therefore not subject to random variation over time. While this assumption may lead to an overestimate of the long-term impact of litter abandonment, the nature of this mechanism was considered to be a realistic simulation of the abandonment process.
- Under the TR option, a minimum treatment threshold of approximately 30% of the remaining naïve (untreated) population per timestep typically resulted in consistent population decline, while TNR required 40% treatment rate to achieve similar results.
- Over a ten year period, sterilizing 40% of the naïve animals during each timestep resulted in a long-term cumulative sterilization rate of 75%.

## Discussion

**Figure 6:**
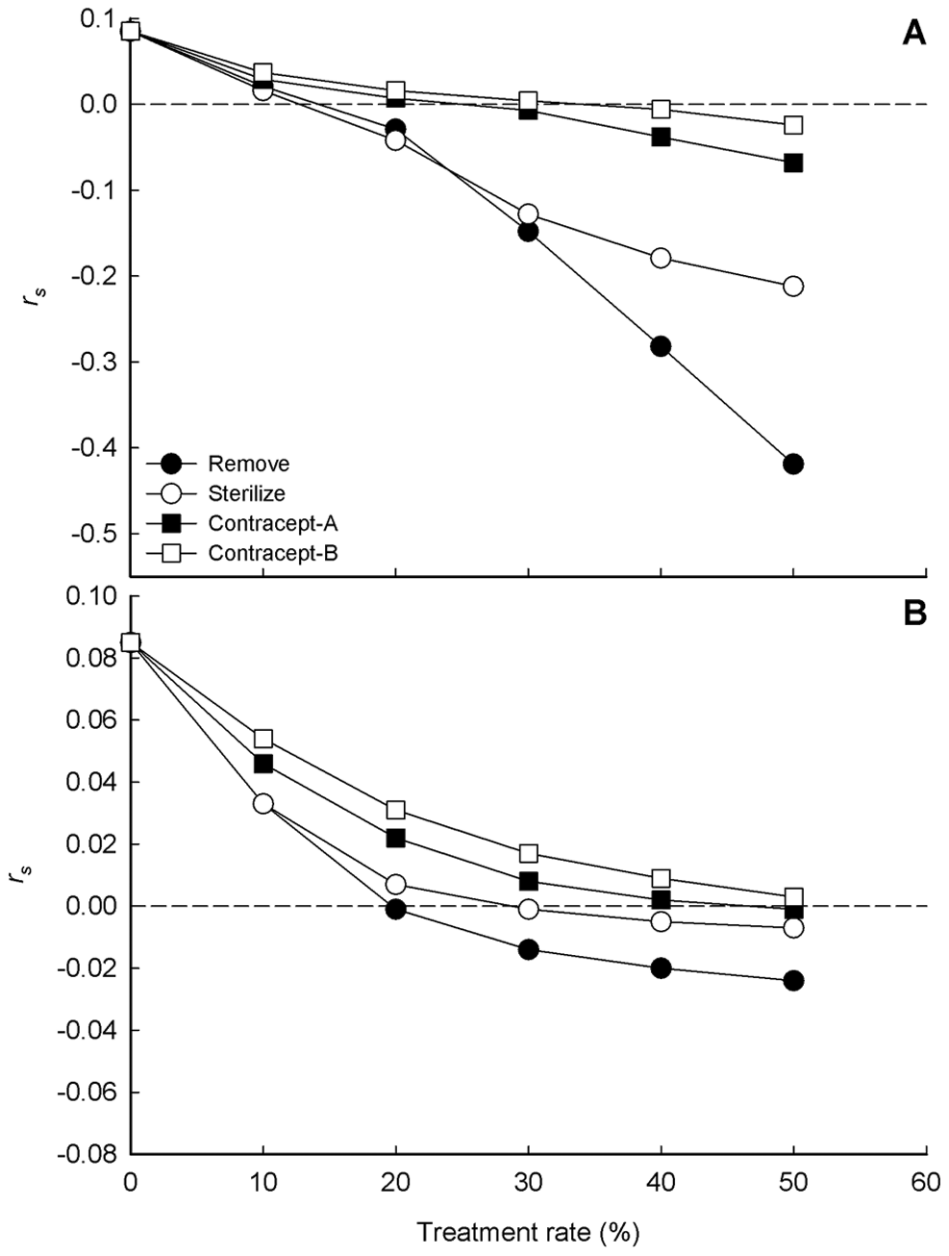
Stochastic population growth rate *r_s_* under different FRC management strategies. (A) Simulated Large Urban populations under conditions of demographic isolation. (B) Simulated Large Urban populations under conditions of demographic connectivity. Treatment rate applies to both kittens and adults. Dashed line indicates the condition where *r_s_* = 0.0.

Insights gained from this detailed modeling effort indicated that temporary contraception options for FRC population management perform nearly as well as TNR in the short term (<5 years), but were less effective than TNR or TR over the longer term (>10 years) (Figure 3). If we consider a negative long-term population growth rate (*r_s_*<0.0) as a generic criterion for population management success, a demographically isolated FRC population is expected to decline in abundance over the long-term if approximately 15% of the population is removed or if 15–20% of the reproductively active population is sterilized, every six months, on a sustained basis. In contrast, approximately 30% of the reproductively active population must be contracepted to achieve the same long-term result (Figure 6A). Successful population management under conditions of demographic connectivity would require removing 20% of the population; or, sterilizing 30% of the untreated segment of the population; or, temporarily contracepting at least 50% of the untreated segment of the population, every six months, on a sustained basis (Figure 6B).

As treatment rates increase, the eventual equilibrium population size will become smaller, or (in isolated populations) the probability of eventual elimination will increase (Figure 4; Tables S4–S15). Substantial time lags exist between initiation of treatment and arrival at a new equilibrium for all treatment options, but time lags would be shorter with increasing treatment rates. Time lags associated with TR are shorter than those associated with sterilization or contraceptive options (Figure 6), because TR not only eliminates the reproductive potential of treated animals, but immediately subtracts them from the population rather than allowing their removal over time through natural mortality.

The biological processes modeled in this study are critical for determining optimal solutions for FRC population management, but economic, social and other considerations also will factor prominently into the final choice(s) among multiple management options. For example, TR may be the most effective method to achieve a rapid and sustained reduction in FRC population size if the ultimate goal is to minimize impacts to associated threatened species. However, additional effort will be required to prevent the newly-vacated space from being re-occupied by reproducing animals. TR options tend to be undertaken by paid municipal staff or contractors, whereas TNR or contraceptive options may be subsidized to some extent by volunteer labor or by charitable contributions to non-profit organizations. Contraceptive methods that can be administered in the field will, at least in principle, be less expensive and time-consuming than surgical sterilization, since the latter requires transportation, clinic space, and veterinary expertise. This alternative also may alleviate ethical concerns over the prospects of euthanizing a large percentage of the individuals removed from the population. However, trapping effort and expense for sterilization-based methods may increase on a per-cat basis as the proportion of non-sterilized target animals declines with treatment over time. Accounting for trapping effort also is important if specific subsets of the population (i.e. adult females) are targeted selectively. Therefore, while beyond the scope of this analysis, a final comparison among management scenarios within a practical decision-making framework ultimately will need to determine the demographic impacts that can be achieved on a per-dollar basis, rather than on a per-procedure basis.

Because of our assumptions underlying male breeding trends, our models indicated that TR or TNR strategies targeting only males have negligible impact on long-term population size, while the same treatments targeting females can achieve more substantial population reductions (Figure 5). This result may be important for designing aggressive treatment programs when finances and personnel resources are limited, and when population reduction is a more immediate priority than other criteria, such as a reduction in hormonally-driven tomcat behaviors.

This study clarifies the importance of realistically accounting for immigration, emigration, owned cat abandonment, and specific modes of density dependence in assessing alternative methods for managing FRC populations (Figure 4). In particular, demographic connectivity with cats in the surrounding environment poses a significant impediment to reducing FRC population size (Figure 6). This suggests that attempts to mitigate contributing human behaviors (e.g., allowing owned cats to roam freely, abandoning unwanted cats) must be implemented concurrently with sound biological management. While a subset of earlier studies of FRC population management have included just one or two of these processes (e.g., [16]–[18] [20]–[22], our study is the only one to incorporate the full range of demographic processes we believe are necessary to evaluate management options in a biologically realistic manner. Similarly, while a selection of previous studies have used simpler modeling tools to evaluate the efficacy of different contraception options on FRC population dynamics [14],[20], our detailed examination of contraception in the context of the biological realism just described yields a more robust comparison among population management alternatives (Figure 4).

This approach to model construction and input parameter estimation generates correspondingly realistic FRC population growth rates that reflect those measured in the field. Therefore, we are not required to follow previous modeling efforts [15],[19] that rely on ecological theory to derive estimates of expected population growth rates. These estimates include *r_m_*, the maximum population growth rate expected under optimal conditions and in the absence of resource limitations [34], which are used to estimate control efforts required to generate negative population growth. Growth rates derived from this theoretical analysis are very often gross overestimates of those expected under realistic conditions. Consequently, the intensity of recommended management (i.e., rate of removal) would be greater than what would come from analysis of more reasonable field data. This may have meaningful implications for designing practical management protocols with constraints on funding, personnel, etc.

All of the strategies that we evaluated had measurable impacts on FRC population size under at least some plausible implementation scenarios (Figure 4; Tables S4–S15). Additional research is needed to better integrate the biological, economic, and sociological considerations in FRC management to provide practical guidance to the cat population management community.

## Supporting Information

Figure S1 **Simulated breeding pattern among adult female free-roaming cat populations.** The graph shows the seasonal pattern of reproductive success based on the six-month timestep featured in all simulations.
(TIF)

Figure S2 **Simulated density dependence in kitten mortality in free-roaming cat populations.**
(TIF)

Figure S3 **Linear regression describing data on return of fertility among female cats treated with the GnRH vaccine GonaCon^TM^.** Data from [12].
(TIF)

Figure S4 **Elasticity of selected demographic parameters in the baseline free-roaming cat population model.** Kittens are defined here as those individuals that are just under six months old and will therefore be able to reproduce in the next timestep, with adults >6 months old. Fecundity is defined as the mean number of female offspring produced each 6-month timestep by either kits or adults.
(TIF)

Table S1 **Litter size distribution used for free-roaming cat population model.**
(DOCX)

Table S2 **Density, initial abundance and carrying capacity estimates for the three population types featured in free-roaming cat population models.**
(DOCX)

Table S3 **Stage-based demographic matrix constructed for elasticity analysis.** Kittens in this table are defined as those individuals that are just under six months old and will therefore be able to reproduce in the next timestep. Fecundity values in the top row describe the number of female kittens that are produced per female and that survive to six months of age. Survival values in the bottom row describe the probability of surviving during a given six-month time interval.
(DOCX)

Table S4 **Full set of scenario results for the Removal management strategy applied to the Large Urban population.** Results in this and each of the following tables include: • r_s_(SD), mean (standard deviation) stochastic growth rate; • P(E), probability of population elimination within the 50 years of the simulation; • T(E), mean time of population elimination among iterations that become extinct; • N_50_(SD), mean (standard deviation) population size at year 50 across all iterations, including those that may result in population elimination.
(DOCX)

Table S5 **Full set of scenario results for the Sterilize management strategy applied to the Large Urban population.** Column heading definitions are identical to those in Table S4.
(DOCX)

Table S6 **Full set of scenario results for the Contracept-A management strategy applied to the Large Urban population.** Column heading definitions are identical to those in Table S4.
(DOCX)

Table S7 **Full set of scenario results for the Contracept-B management strategy applied to the Large Urban population.** Column heading definitions are identical to those in Table S4.
(DOCX)

Table S8 **Full set of scenario results for the Removal management strategy applied to the Small Urban population.** Column heading definitions are identical to those in Table S4.
(DOCX)

Table S9 **Full set of scenario results for the Sterilize management strategy applied to the Small Urban population.** Column heading definitions are identical to those in Table S4.
(DOCX)

Table S10 **Full set of scenario results for the Contracept-A management strategy applied to the Small Urban population.** Column heading definitions are identical to those in Table S4.
(DOCX)

Table S11 **Full set of scenario results for the Contracept-B management strategy applied to the Small Urban population.** Column heading definitions are identical to those in Table S4.
(DOCX)

Table S12 **Full set of scenario results for the Removal management strategy applied to the Rural population.** Column heading definitions are identical to those in Table S4.
(DOCX)

Table S13 **Full set of scenario results for the Sterilize management strategy applied to the Rural population.** Column heading definitions are identical to those in Table S4.
(DOCX)

Table S14 **Full set of scenario results for the Contracept-A management strategy applied to the Rural population.** Column heading definitions are identical to those in Table S4.
(DOCX)

Table S15 **Full set of scenario results for the Contracept-B management strategy applied to the Rural population.** Column heading definitions are identical to those in Table S4.
(DOCX)

File S1 **Main supporting information file.** This file includes additional materials and methods (model structure and input data), additional results (elasticity analysis), and additional references.
(DOCX)
