# Correction: Simulating Free-Roaming Cat Population Management Options in Open Demographic Environments

**DOI:** 10.1371/journal.pone.0119390

**Published:** 2015-03-16

**Authors:** The PLOS ONE Staff

There is an error in the third sentence of the Abstract. The definition of TR should be trap-remove, not trap-return. The complete, correct sentence is: Previous analyses that use simulation modeling tools to evaluate alternative management methods have focused on relative efficacy of removal (or trap-remove, TR), typically involving euthanasia, and sterilization (or trap-neuter-return, TNR) in demographically isolated populations.

## References

[1] Miller PS, Boone JD, Briggs JR, Lawler DF, Levy JK, Nutter FB, et al (2014) Simulating Free-Roaming Cat Population Management Options in Open Demographic Environments. PLoS ONE 9(11): e113553 doi:10.1371/journal.pone.0113553 25426960 10.1371/journal.pone.0113553 PMC4245120

