# Supplementary figures and images for: Simulating Free-Roaming Cat Population Management Options in Open Demographic Environments
Source: PLoS One. 2014 Nov 26;9(11):e113553. doi: 10.1371/journal.pone.0113553 (PMC4245120; doi:10.1371/journal.pone.0113553)

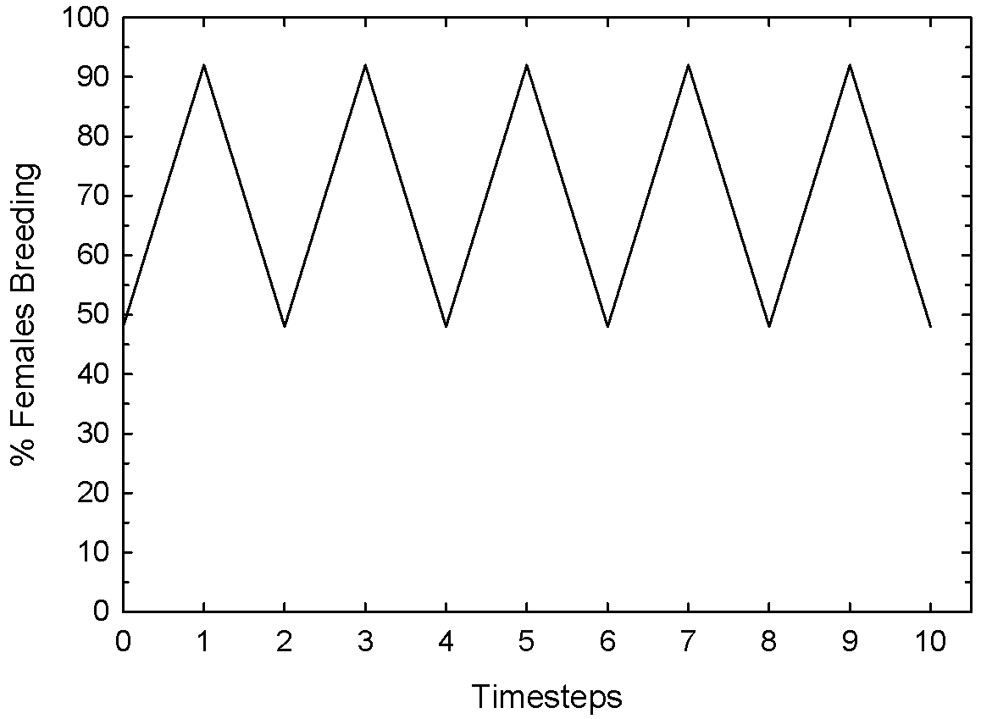

Supplement: Figure S1 — Simulated breeding pattern among adult female free-roaming cat populations. The graph shows the seasonal pattern of reproductive success based on the six-month timestep featured in all simulations. (TIF) [file pone.0113553.s001.tif]

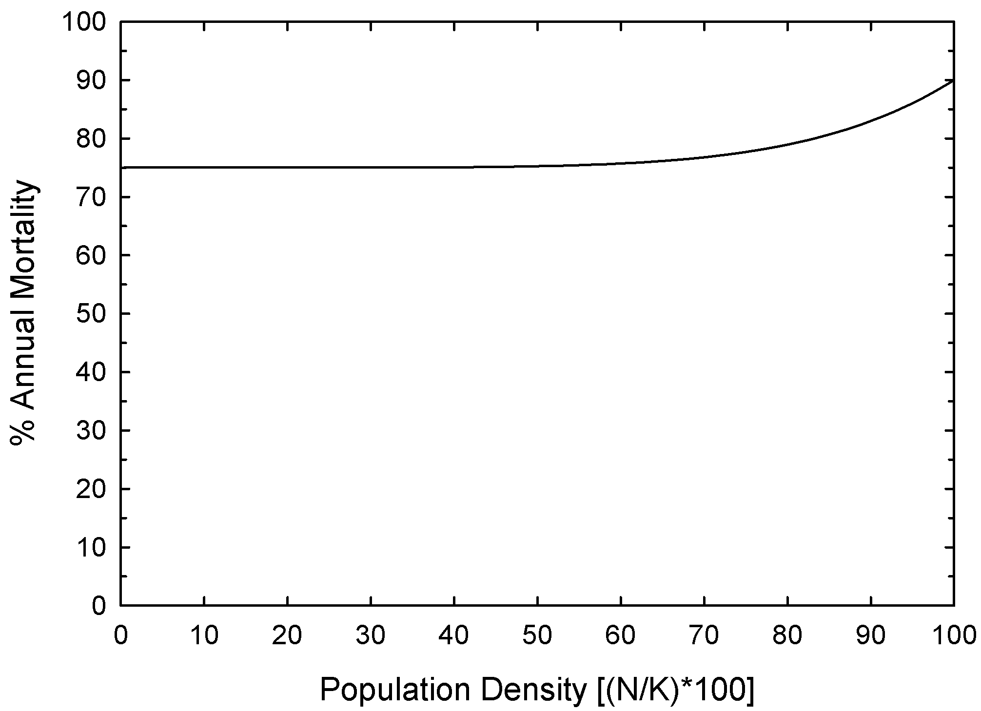

Supplement: Figure S2 — Simulated density dependence in kitten mortality in free-roaming cat populations. (TIF) [file pone.0113553.s002.tif]

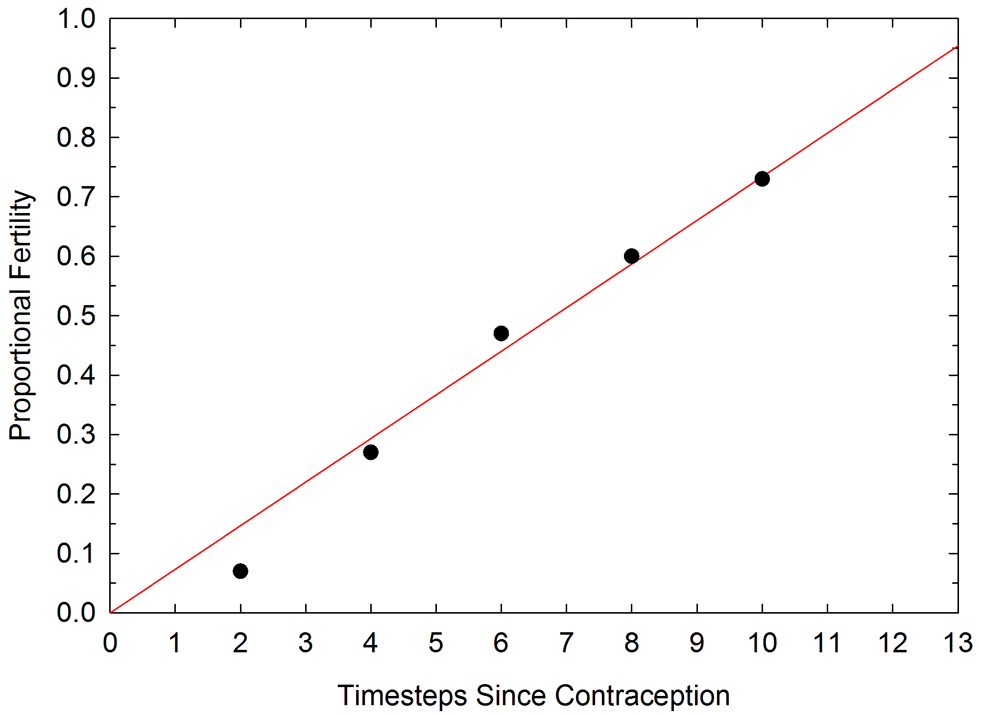

Supplement: Figure S3 — Linear regression describing data on return of fertility among female cats treated with the GnRH vaccine GonaConTM. Data from [12]. (TIF) [file pone.0113553.s003.tif]

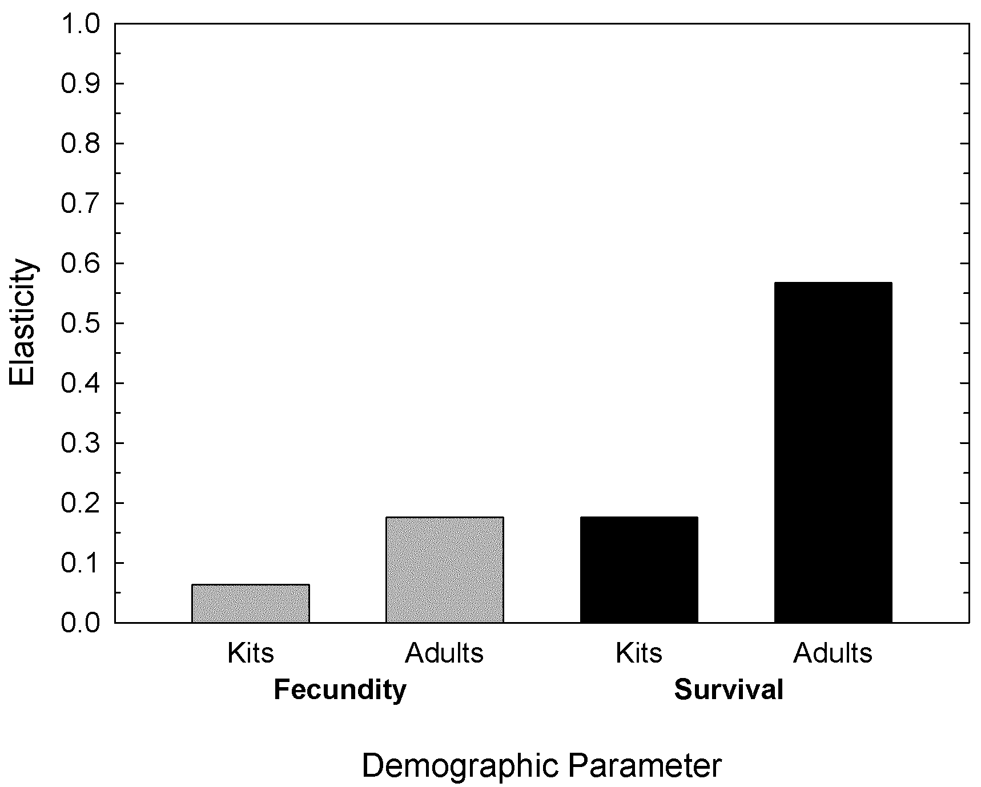

Supplement: Figure S4 — Elasticity of selected demographic parameters in the baseline free-roaming cat population model. Kittens are defined here as those individuals that are just under six months old and will therefore be able to reproduce in the next timestep, with adults >6 months old. Fecundity is defined as the mean number of female offspring produced each 6-month timestep by either kits or adults. (TIF) [file pone.0113553.s004.tif]
