# Supplementary material for: Simulating Free-Roaming Cat Population Management Options in Open Demographic Environments
Source: PLoS One. 2014 Nov 26;9(11):e113553. doi: 10.1371/journal.pone.0113553 (PMC4245120; doi:10.1371/journal.pone.0113553)
Supplement: Table S1 — Litter size distribution used for free-roaming cat population model. (DOCX) [file pone.0113553.s005.docx]

| Litter Size | Probability (%) |
| --- | --- |
| 1 | 3.0 |
| 2 | 5.0 |
| 3 | 50.0 |
| 4 | 30.0 |
| 5 | 8.0 |
| 6 | 4.0 |
