# Supplementary material for: Simulating Free-Roaming Cat Population Management Options in Open Demographic Environments
Source: PLoS One. 2014 Nov 26;9(11):e113553. doi: 10.1371/journal.pone.0113553 (PMC4245120; doi:10.1371/journal.pone.0113553)
Supplement: Table S2 — Density, initial abundance and carrying capacity estimates for the three population types featured in free-roaming cat population models. (DOCX) [file pone.0113553.s006.docx]

| Population Type | Density (#/km^2^) | Initial Abundance | Carrying Capacity |
| --- | --- | --- | --- |
| Large Urban | 400 | 200 | 200 |
| Small Urban | 200 | 100 | 100 |
| Rural | 50 | 25 | 25 |
