# Supplementary material for: Simulating Free-Roaming Cat Population Management Options in Open Demographic Environments
Source: PLoS One. 2014 Nov 26;9(11):e113553. doi: 10.1371/journal.pone.0113553 (PMC4245120; doi:10.1371/journal.pone.0113553)
Supplement: Table S3 — Stage-based demographic matrix constructed for elasticity analysis. Kittens in this table are defined as those individuals that are just under six months old and will therefore be able to reproduce in the next timestep. Fecundity values in the top row describe the number of female kittens that are produced per female and that survive to six months of age. Survival values in the bottom row describe the probability of surviving during a given six-month time interval. (DOCX) [file pone.0113553.s007.docx]

|  | Kittens (6mos) | Adults (12mos) |
| --- | --- | --- |
| Kittens | *F*_0_ = 0.304 | *F*_1_ = 0.304 |
| Adults | *S*_0_ = 0.948 | *S*_1_ = 0.948 |
