# Supplementary material for: Simulating Free-Roaming Cat Population Management Options in Open Demographic Environments
Source: PLoS One. 2014 Nov 26;9(11):e113553. doi: 10.1371/journal.pone.0113553 (PMC4245120; doi:10.1371/journal.pone.0113553)
Supplement: Table S4 — Full set of scenario results for the Removal management strategy applied to the Large Urban population. Results in this and each of the following tables include: • rs(SD), mean (standard deviation) stochastic growth rate; • P(E), probability of population elimination within the 50 years of the simulation; • T(E), mean time of population elimination among iterations that become extinct; • N50(SD), mean (standard deviation) population size at year 50 across all iterations, including those that may result in population elimination. (DOCX) [file pone.0113553.s008.docx]

| **Scenario** | | **r_s_ (SD)** | **P(E)** | **T(E)** | **N_50_ (SD)** |
| --- | --- | --- | --- | --- | --- |
| Baseline | | 0.085 (0.144) | 0.000 |  | 193 (10) |
| Abandon/Dispersal | Kits 10% | 0.073 (0.135) | 0.000 |  | 191 (10) |
|  | Kits 20% | 0.059 (0.126) | 0.000 |  | 191 (11) |
|  | Kits 30% | 0.047 (0.117) | 0.000 |  | 189 (12) |
|  | Kits 40% | 0.035 (0.107) | 0.000 |  | 186 (13) |
|  | Kits 50% | 0.021 (0.098) | 0.000 |  | 183 (15) |
|  | Adults 10% | 0.044 (0.165) | 0.000 |  | 180 (17) |
|  | Adults 20% | 0.012 (0.185) | 0.000 |  | 154 (27) |
|  | Adults 30% | -0.005 (0.020) | 0.000 |  | 98 (33) |
|  | Adults 40% | -0.014 (0.227) | 0.000 |  | 44 (18) |
|  | Adults 50% | -0.019 (0.264) | 0.000 |  | 26 (9) |
|  | Both 10% | 0.033 (0.154) | 0.000 |  | 176 (19) |
|  | Both 20% | -0.001 (0.160) | 0.000 |  | 131 (31) |
|  | Both 30% | -0.014 (0.175) | 0.000 |  | 47 (14) |
|  | Both 40% | -0.020 (0.210) | 0.000 |  | 25 (7) |
|  | Both 50% | -0.024 (0.250) | 0.000 |  | 17 (5) |
| Dispersal | Kits 10% | 0.060 (0.142) | 0.000 |  | 188 (14) |
|  | Kits 20% | 0.047 (0.133) | 0.000 |  | 185 (15) |
|  | Kits 30% | 0.034 (0.124) | 0.000 |  | 183 (16) |
|  | Kits 40% | 0.021 (0.116) | 0.000 |  | 178 (18) |
|  | Kits 50% | 0.010 (0.106) | 0.000 |  | 170 (21) |
|  | Adults 10% | 0.034 (0.171) | 0.000 |  | 173 (21) |
|  | Adults 20% | 0.005 (0.188) | 0.000 |  | 136 (35) |
|  | Adults 30% | -0.016 (0.225) | 0.007 | 32.6 | 40 (26) |
|  | Adults 40% | -0.025 (0.311) | 0.048 | 25.8 | 15 (8) |
|  | Adults 50% | -0.029 (0.391) | 0.121 | 16.5 | 9 (5) |
|  | Both 10% | 0.023 (0.160) | 0.000 |  | 169 (23) |
|  | Both 20% | -0.008 (0.169) | 0.000 |  | 82 (40) |
|  | Both 30% | -0.025 (0.251) | 0.027 | 28.0 | 17 (8) |
|  | Both 40% | -0.031 (0.341) | 0.113 | 16.9 | 9 (4) |
|  | Both 50% | -0.039 (0.418) | 0.197 | 9.0 | 7 (3) |
| Abandon | Kits 10% | 0.070 (0.141) | 0.000 |  | 191 (12) |
|  | Kits 20% | 0.056 (0.132) | 0.000 |  | 189 (12) |
|  | Kits 30% | 0.044 (0.122) | 0.000 |  | 187 (14) |
|  | Kits 40% | 0.030 (0.112) | 0.000 |  | 184 (15) |
|  | Kits 50% | 0.018 (0.102) | 0.000 |  | 180 (16) |
|  | Adults 10% | 0.042 (0.171) | 0.000 |  | 178 (18) |
|  | Adults 20% | 0.010 (0.190) | 0.000 |  | 149 (27) |
|  | Adults 30% | -0.008 (0.207) | 0.000 |  | 80 (34) |
|  | Adults 40% | -0.019 (0.243) | 0.000 |  | 30 (14) |
|  | Adults 50% | -0.024 (0.284) | 0.000 |  | 17 (7) |
|  | Both 10% | 0.030 (0.159) | 0.000 |  | 175 (19) |
|  | Both 20% | -0.002 (0.164) | 0.000 |  | 122 (34) |
|  | Both 30% | -0.018 (0.189) | 0.000 |  | 32 (12) |
|  | Both 40% | -0.025 (0.227) | 0.000 |  | 16 (5) |
|  | Both 50% | -0.029 (0.262) | 0.000 |  | 11 (3) |
| Isolated | Kits 10% | 0.056 (0.148) | 0.000 |  | 187 (14) |
|  | Kits 20% | 0.044 (0.139) | 0.000 |  | 184 (15) |
|  | Kits 30% | 0.031 (0.131) | 0.000 |  | 181 (17) |
|  | Kits 40% | 0.019 (0.121) | 0.000 |  | 176 (19) |
|  | Kits 50% | 0.007 (0.111) | 0.000 |  | 163 (26) |
|  | Adults 10% | 0.032 (0.176) | 0.000 |  | 170 (22) |
|  | Adults 20% | 0.002 (0.195) | 0.010 | 33.2 | 123 (44) |
|  | Adults 30% | -0.064 (0.256) | 0.908 | 27.0 | 2 (10) |
|  | Adults 40% | -0.159 (0.299) | 1.000 | 12.5 |  |
|  | Adults 50% | -0.261 (0.345) | 1.000 | 7.8 |  |
|  | Both 10% | 0.021 (0.166) | 0.000 |  | 166 (23) |
|  | Both 20% | -0.029 (0.192) | 0.417 | 35.4 | 29 (39) |
|  | Both 30% | -0.148 (0.244) | 1.000 | 13.8 |  |
|  | Both 40% | -0.282 (0.276) | 1.000 | 7.5 |  |
|  | Both 50% | -0.427 (0.318) | 1.000 | 5.1 |  |
