# Supplementary material for: Simulating Free-Roaming Cat Population Management Options in Open Demographic Environments
Source: PLoS One. 2014 Nov 26;9(11):e113553. doi: 10.1371/journal.pone.0113553 (PMC4245120; doi:10.1371/journal.pone.0113553)
Supplement: Table S5 — Full set of scenario results for the Sterilize management strategy applied to the Large Urban population. Column heading definitions are identical to those in Table S4. (DOCX) [file pone.0113553.s009.docx]

| **Scenario** | | **r_s_ (SD)** | **P(E)** | **T(E)** | **N_50_ (SD)** |
| --- | --- | --- | --- | --- | --- |
| Baseline | | 0.085 (0.144) | 0.000 |  | 193 (10) |
| Abandon/Dispersal | Kits 10% | 0.073 (0.135) | 0.000 |  | 191 (11) |
|  | Kits 20% | 0.063 (0.128) | 0.000 |  | 191 (12) |
|  | Kits 30% | 0.051 (0.120) | 0.000 |  | 189 (12) |
|  | Kits 40% | 0.040 (0.113) | 0.000 |  | 187 (13) |
|  | Kits 50% | 0.028 (0.105) | 0.000 |  | 184 (14) |
|  | Adults 10% | 0.041 (0.113) | 0.000 |  | 187 (14) |
|  | Adults 20% | 0.015 (0.094) | 0.000 |  | 177 (18) |
|  | Adults 30% | 0.004 (0.084) | 0.000 |  | 159 (24) |
|  | Adults 40% | -0.001 (0.079) | 0.000 |  | 137 (27) |
|  | Adults 50% | -0.003 (0.076) | 0.000 |  | 120 (24) |
|  | Both 10% | 0.033 (0.107) | 0.000 |  | 186 (13) |
|  | Both 20% | 0.007 (0.087) | 0.000 |  | 166 (22) |
|  | Both 30% | -0.001 (0.078) | 0.000 |  | 129 (25) |
|  | Both 40% | -0.005 (0.074) | 0.000 |  | 100 (19) |
|  | Both 50% | -0.007 (0.073) | 0.000 |  | 85 (14) |
| Dispersal | Kits 10% | 0.061 (0.144) | 0.000 |  | 187 (15) |
|  | Kits 20% | 0.049 (0.135) | 0.000 |  | 186 (15) |
|  | Kits 30% | 0.038 (0.128) | 0.000 |  | 183 (16) |
|  | Kits 40% | 0.027 (0.121) | 0.000 |  | 180 (18) |
|  | Kits 50% | 0.016 (0.113) | 0.000 |  | 171 (21) |
|  | Adults 10% | 0.027 (0.120) | 0.000 |  | 179 (18) |
|  | Adults 20% | 0.004 (0.104) | 0.000 |  | 148 (35) |
|  | Adults 30% | -0.007 (0.106) | 0.000 |  | 85 (38) |
|  | Adults 40% | -0.012 (0.115) | 0.000 |  | 51 (23) |
|  | Adults 50% | -0.015 (0.123) | 0.000 |  | 39 (15) |
|  | Both 10% | 0.020 (0.115) | 0.000 |  | 176 (20) |
|  | Both 20% | -0.003 (0.102) | 0.000 |  | 111 (41) |
|  | Both 30% | -0.012 (0.112) | 0.001 | 64.6 | 51 (21) |
|  | Both 40% | -0.016 (0.121) | 0.000 |  | 36 (11) |
|  | Both 50% | -0.017 (0.129) | 0.001 | 61.2 | 32 (9) |
| Abandon | Kits 10% | 0.071 (0.141) | 0.000 |  | 191 (12) |
|  | Kits 20% | 0.058 (0.133) | 0.000 |  | 190 (12) |
|  | Kits 30% | 0.046 (0.125) | 0.000 |  | 188 (13) |
|  | Kits 40% | 0.034 (0.117) | 0.000 |  | 185 (14) |
|  | Kits 50% | 0.022 (0.108) | 0.000 |  | 179 (18) |
|  | Adults 10% | 0.037 (0.118) | 0.000 |  | 184 (14) |
|  | Adults 20% | 0.011 (0.099) | 0.000 |  | 171 (22) |
|  | Adults 30% | 0.001 (0.089) | 0.000 |  | 143 (31) |
|  | Adults 40% | -0.003 (0.085) | 0.000 |  | 113 (29) |
|  | Adults 50% | -0.006 (0.082) | 0.000 |  | 89 (24) |
|  | Both 10% | 0.028 (0.112) | 0.000 |  | 182 (16) |
|  | Both 20% | 0.004 (0.091) | 0.000 |  | 153 (29) |
|  | Both 30% | -0.005 (0.082) | 0.000 |  | 98 (27) |
|  | Both 40% | -0.009 (0.080) | 0.000 |  | 66 (15) |
|  | Both 50% | -0.011 (0.079) | 0.000 |  | 53 (9) |
| Isolated | Kits 10% | 0.058 (0.149) | 0.000 |  | 187 (14) |
|  | Kits 20% | 0.045 (0.141) | 0.000 |  | 183 (16) |
|  | Kits 30% | 0.033 (0.132) | 0.000 |  | 182 (18) |
|  | Kits 40% | 0.022 (0.125) | 0.000 |  | 174 (21) |
|  | Kits 50% | 0.011 (0.116) | 0.000 |  | 160 (28) |
|  | Adults 10% | 0.024 (0.125) | 0.000 |  | 176 (21) |
|  | Adults 20% | -0.005 (0.119) | 0.132 | 69.5 | 106 (62) |
|  | Adults 30% | -0.078 (0.164) | 0.959 | 46.0 | 2 (12) |
|  | Adults 40% | -0.142 (0.208) | 1.000 | 27.8 |  |
|  | Adults 50% | -0.185 (0.267) | 1.000 | 21.5 |  |
|  | Both 10% | 0.016 (0.120) | 0.000 |  | 168 (25) |
|  | Both 20% | -0.042 (0.141) | 0.701 | 60 | 19 (40) |
|  | Both 30% | -0.128 (0.191) | 1.000 | 30.9 |  |
|  | Both 40% | -0.179 (0.250) | 1.000 | 22.1 |  |
|  | Both 50% | -0.212 (0.316) | 1.000 | 18.8 |  |
