# Supplementary material for: Simulating Free-Roaming Cat Population Management Options in Open Demographic Environments
Source: PLoS One. 2014 Nov 26;9(11):e113553. doi: 10.1371/journal.pone.0113553 (PMC4245120; doi:10.1371/journal.pone.0113553)
Supplement: Table S6 — Full set of scenario results for the Contracept-A management strategy applied to the Large Urban population. Column heading definitions are identical to those in Table S4. (DOCX) [file pone.0113553.s010.docx]

| **Scenario** | | **r_s_ (SD)** | **P(E)** | **T(E)** | **N_50_ (SD)** |
| --- | --- | --- | --- | --- | --- |
| Baseline | | 0.085 (0.144) | 0.000 |  | 193 (10) |
| Abandon/Dispersal | Kits 10% | 0.078 (0.139) | 0.000 |  | 193 (10) |
|  | Kits 20% | 0.071 (0.133) | 0.000 |  | 192 (11) |
|  | Kits 30% | 0.063 (0.128) | 0.000 |  | 191 (11) |
|  | Kits 40% | 0.056 (0.123) | 0.000 |  | 191 (11) |
|  | Kits 50% | 0.049 (0.118) | 0.000 |  | 190 (12) |
|  | Adults 10% | 0.052 (0.120) | 0.000 |  | 190 (12) |
|  | Adults 20% | 0.029 (0.104) | 0.000 |  | 186 (13) |
|  | Adults 30% | 0.016 (0.095) | 0.000 |  | 179 (17) |
|  | Adults 40% | 0.008 (0.088) | 0.000 |  | 172 (19) |
|  | Adults 50% | 0.003 (0.084) | 0.000 |  | 163 (22) |
|  | Both 10% | 0.046 (0.116) | 0.000 |  | 188 (13) |
|  | Both 20% | 0.022 (0.099) | 0.000 |  | 183 (16) |
|  | Both 30% | 0.008 (0.088) | 0.000 |  | 173 (18) |
|  | Both 40% | 0.002 (0.082) | 0.000 |  | 159 (22) |
|  | Both 50% | -0.001 (0.077) | 0.000 |  | 141 (23) |
| Dispersal | Kits 10% | 0.065 (0.146) | 0.000 |  | 189 (13) |
|  | Kits 20% | 0.057 (0.141) | 0.000 |  | 188 (14) |
|  | Kits 30% | 0.050 (0.136) | 0.000 |  | 186 (15) |
|  | Kits 40% | 0.043 (0.132) | 0.000 |  | 185 (15) |
|  | Kits 50% | 0.037 (0.127) | 0.000 |  | 183 (16) |
|  | Adults 10% | 0.036 (0.127) | 0.000 |  | 183 (16) |
|  | Adults 20% | 0.016 (0.111) | 0.000 |  | 174 (21) |
|  | Adults 30% | 0.006 (0.103) | 0.000 |  | 162 (26) |
|  | Adults 40% | -0.000 (0.098) | 0.000 |  | 139 (33) |
|  | Adults 50% | -0.005 (0.098) | 0.000 |  | 102 (36) |
|  | Both 10% | 0.032 (0.123) | 0.000 |  | 182 (17) |
|  | Both 20% | 0.011 (0.107) | 0.000 |  | 168 (23) |
|  | Both 30% | 0.000 (0.097) | 0.000 |  | 142 (32) |
|  | Both 40% | -0.007 (0.097) | 0.000 |  | 88 (32) |
|  | Both 50% | -0.012 (0.103) | 0.000 |  | 55 (20) |
| Abandon | Kits 10% | 0.074 (0.144) | 0.000 |  | 192 (11) |
|  | Kits 20% | 0.067 (0.139) | 0.000 |  | 191 (12) |
|  | Kits 30% | 0.059 (0.133) | 0.000 |  | 189 (12) |
|  | Kits 40% | 0.051 (0.128) | 0.000 |  | 187 (13) |
|  | Kits 50% | 0.044 (0.122) | 0.000 |  | 187 (13) |
|  | Adults 10% | 0.047 (0.125) | 0.000 |  | 187 (13) |
|  | Adults 20% | 0.025 (0.109) | 0.000 |  | 181 (16) |
|  | Adults 30% | 0.012 (0.099) | 0.000 |  | 174 (20) |
|  | Adults 40% | 0.005 (0.093) | 0.000 |  | 165 (22) |
|  | Adults 50% | 0.001 (0.088) | 0.000 |  | 151 (26) |
|  | Both 10% | 0.042 (0.121) | 0.000 |  | 187 (13) |
|  | Both 20% | 0.018 (0.103) | 0.000 |  | 178 (18) |
|  | Both 30% | 0.006 (0.092) | 0.000 |  | 166 (22) |
|  | Both 40% | -0.000 (0.085) | 0.000 |  | 145 (26) |
|  | Both 50% | -0.004 (0.081) | 0.000 |  | 112 (26) |
| Isolated | Kits 10% | 0.062 (0.152) | 0.000 |  | 187 (14) |
|  | Kits 20% | 0.054 (0.147) | 0.000 |  | 186 (14) |
|  | Kits 30% | 0.047 (0.142) | 0.000 |  | 185 (15) |
|  | Kits 40% | 0.040 (0.137) | 0.000 |  | 184 (15) |
|  | Kits 50% | 0.033 (0.133) | 0.000 |  | 181 (16) |
|  | Adults 10% | 0.033 (0.132) | 0.000 |  | 181 (18) |
|  | Adults 20% | 0.014 (0.117) | 0.000 |  | 168 (24) |
|  | Adults 30% | 0.003 (0.109) | 0.001 | 62.0 | 147 (35) |
|  | Adults 40% | -0.008 (0.111) | 0.057 | 76.6 | 94 (53) |
|  | Adults 50% | -0.031 (0.131) | 0.432 | 71.6 | 22 (32) |
|  | Both 10% | 0.029 (0.129) | 0.000 |  | 179 (18) |
|  | Both 20% | 0.007 (0.112) | 0.000 |  | 160 (28) |
|  | Both 30% | -0.007 (0.109) | 0.035 | 74.0 | 100 (51) |
|  | Both 40% | -0.038 (0.136) | 0.592 | 72.4 | 12 (21) |
|  | Both 50% | -0.068 (0.160) | 0.975 | 56.9 | 1 (2) |
