# Supplementary material for: Simulating Free-Roaming Cat Population Management Options in Open Demographic Environments
Source: PLoS One. 2014 Nov 26;9(11):e113553. doi: 10.1371/journal.pone.0113553 (PMC4245120; doi:10.1371/journal.pone.0113553)
Supplement: Table S7 — Full set of scenario results for the Contracept-B management strategy applied to the Large Urban population. Column heading definitions are identical to those in Table S4. (DOCX) [file pone.0113553.s011.docx]

| **Scenario** | | **r_s_ (SD)** | **P(E)** | **T(E)** | **N_50_ (SD)** |
| --- | --- | --- | --- | --- | --- |
| Baseline | | 0.085 (0.144) | 0.000 |  | 193 (10) |
| Abandon/Dispersal | Kits 10% | 0.079 (0.139) | 0.000 |  | 192 (11) |
|  | Kits 20% | 0.074 (0.136) | 0.000 |  | 192 (11) |
|  | Kits 30% | 0.068 (0.132) | 0.000 |  | 192 (10) |
|  | Kits 40% | 0.063 (0.128) | 0.000 |  | 191 (11) |
|  | Kits 50% | 0.057 (0.125) | 0.000 |  | 191 (11) |
|  | Adults 10% | 0.058 (0.124) | 0.000 |  | 190 (12) |
|  | Adults 20% | 0.038 (0.110) | 0.000 |  | 189 (13) |
|  | Adults 30% | 0.025 (0.101) | 0.000 |  | 183 (15) |
|  | Adults 40% | 0.015 (0.094) | 0.000 |  | 179 (16) |
|  | Adults 50% | 0.009 (0.088) | 0.000 |  | 175 (19) |
|  | Both 10% | 0.054 (0.122) | 0.000 |  | 189 (12) |
|  | Both 20% | 0.031 (0.106) | 0.000 |  | 186 (13) |
|  | Both 30% | 0.017 (0.096) | 0.000 |  | 179 (16) |
|  | Both 40% | 0.009 (0.088) | 0.000 |  | 174 (18) |
|  | Both 50% | 0.003 (0.083) | 0.000 |  | 164 (20) |
| Dispersal | Kits 10% | 0.066 (0.147) | 0.000 |  | 189 (13) |
|  | Kits 20% | 0.060 (0.140) | 0.000 |  | 188 (13) |
|  | Kits 30% | 0.056 (0.141) | 0.000 |  | 188 (14) |
|  | Kits 40% | 0.051 (0.138) | 0.000 |  | 187 (14) |
|  | Kits 50% | 0.045 (0.133) | 0.000 |  | 185 (15) |
|  | Adults 10% | 0.044 (0.131) | 0.000 |  | 185 (15) |
|  | Adults 20% | 0.025 (0.118) | 0.000 |  | 180 (17) |
|  | Adults 30% | 0.013 (0.118) | 0.000 |  | 170 (21) |
|  | Adults 40% | 0.005 (0.102) | 0.000 |  | 160 (26) |
|  | Adults 50% | 0.000 (0.097) | 0.000 |  | 142 (34) |
|  | Both 10% | 0.039 (0.129) | 0.000 |  | 184 (15) |
|  | Both 20% | 0.019 (0.114) | 0.000 |  | 176 (19) |
|  | Both 30% | 0.007 (0.104) | 0.000 |  | 164 (25) |
|  | Both 40% | 0.000 (0.097) | 0.000 |  | 143 (32) |
|  | Both 50% | -0.004 (0.095) | 0.000 |  | 108 (35) |
| Abandon | Kits 10% | 0.077 (0.145) | 0.000 |  | 191 (12) |
|  | Kits 20% | 0.070 (0.141) | 0.000 |  | 191 (11) |
|  | Kits 30% | 0.065 (0.138) | 0.000 |  | 190 (12) |
|  | Kits 40% | 0.059 (0.134) | 0.000 |  | 190 (12) |
|  | Kits 50% | 0.054 (0.130) | 0.000 |  | 189 (12) |
|  | Adults 10% | 0.054 (0.130) | 0.000 |  | 189 (13) |
|  | Adults 20% | 0.034 (0.116) | 0.000 |  | 184 (15) |
|  | Adults 30% | 0.020 (0.106) | 0.000 |  | 180 (17) |
|  | Adults 40% | 0.012 (0.099) | 0.000 |  | 174 (19) |
|  | Adults 50% | 0.006 (0.092) | 0.000 |  | 167 (22) |
|  | Both 10% | 0.050 (0.127) | 0.000 |  | 188 (13) |
|  | Both 20% | 0.027 (0.111) | 0.000 |  | 182 (16) |
|  | Both 30% | 0.014 (0.100) | 0.000 |  | 176 (18) |
|  | Both 40% | 0.005 (0.092) | 0.000 |  | 166 (21) |
|  | Both 50% | 0.001 (0.086) | 0.000 |  | 152 (25) |
| Isolated | Kits 10% | 0.064 (0.153) | 0.000 |  | 187 (14) |
|  | Kits 20% | 0.058 (0.150) | 0.000 |  | 187 (15) |
|  | Kits 30% | 0.053 (0.146) | 0.000 |  | 186 (15) |
|  | Kits 40% | 0.048 (0.142) | 0.000 |  | 184 (15) |
|  | Kits 50% | 0.043 (0.140) | 0.000 |  | 183 (16) |
|  | Adults 10% | 0.040 (0.137) | 0.000 |  | 182 (17) |
|  | Adults 20% | 0.022 (0.123) | 0.000 |  | 176 (20) |
|  | Adults 30% | 0.010 (0.114) | 0.000 |  | 165 (26) |
|  | Adults 40% | 0.002 (0.107) | 0.001 |  | 148 (34) |
|  | Adults 50% | -0.005 (0.107) | 0.031 | 37.8 | 108 (50) |
|  | Both 10% | 0.037 (0.134) | 0.000 |  | 182 (17) |
|  | Both 20% | 0.016 (0.120) | 0.000 |  | 171 (22) |
|  | Both 30% | 0.004 (0.109) | 0.002 | 28.0 | 153 (31) |
|  | Both 40% | -0.006 (0.106) | 0.021 | 40.4 | 105 (48) |
|  | Both 50% | -0.024 (0.122) | 0.265 | 38.8 | 33 (37) |
