# Supplementary material for: Simulating Free-Roaming Cat Population Management Options in Open Demographic Environments
Source: PLoS One. 2014 Nov 26;9(11):e113553. doi: 10.1371/journal.pone.0113553 (PMC4245120; doi:10.1371/journal.pone.0113553)
Supplement: Table S8 — Full set of scenario results for the Removal management strategy applied to the Small Urban population. Column heading definitions are identical to those in Table S4. (DOCX) [file pone.0113553.s012.docx]

| **Scenario** | | **r_s_ (SD)** | **P(E)** | **T(E)** | **N_50_ (SD)** |
| --- | --- | --- | --- | --- | --- |
| Baseline | | 0.099 (0.143) | 0.000 |  | 98 (5) |
| Abandon/Dispersal | Kits 10% | 0.087 (0.133) | 0.000 |  | 98 (4) |
|  | Kits 20% | 0.074 (0.124) | 0.000 |  | 97 (5) |
|  | Kits 30% | 0.061 (0.115) | 0.000 |  | 97 (5) |
|  | Kits 40% | 0.049 (0.105) | 0.000 |  | 96 (5) |
|  | Kits 50% | 0.036 (0.096) | 0.000 |  | 95 (6) |
|  | Adults 10% | 0.054 (0.165) | 0.000 |  | 92 (8) |
|  | Adults 20% | 0.021 (0.189) | 0.000 |  | 81 (12) |
|  | Adults 30% | 0.000 (0.210) | 0.000 |  | 60 (16) |
|  | Adults 40% | -0.009 (0.236) | 0.000 |  | 35 (13) |
|  | Adults 50% | -0.014 (0.273) | 0.000 |  | 22 (8) |
|  | Both 10% | 0.043 (0.155) | 0.000 |  | 91 (8) |
|  | Both 20% | 0.005 (0.166) | 0.000 |  | 74 (13) |
|  | Both 30% | -0.009 (0.182) | 0.000 |  | 37 (11) |
|  | Both 40% | -0.015 (0.215) | 0.000 |  | 20 (5) |
|  | Both 50% | -0.019 (0.254) | 0.000 |  | 14 (4) |
| Dispersal | Kits 10% | 0.059 (0.147) | 0.000 |  | 94 (7) |
|  | Kits 20% | 0.046 (0.138) | 0.000 |  | 92 (8) |
|  | Kits 30% | 0.034 (0.130) | 0.000 |  | 91 (9) |
|  | Kits 40% | 0.022 (0.121) | 0.000 |  | 88 (10) |
|  | Kits 50% | 0.010 (0.110) | 0.000 |  | 84 (12) |
|  | Adults 10% | 0.033 (0.177) | 0.000 |  | 86 (11) |
|  | Adults 20% | 0.005 (0.201) | 0.001 | 30.7 | 65 (19) |
|  | Adults 30% | -0.016 (0.269) | 0.075 | 28.2 | 19 (16) |
|  | Adults 40% | -0.022 (0.362) | 0.235 | 16.7 | 7 (5) |
|  | Adults 50% | -0.027 (0.433) | 0.383 | 9.4 | 4 (3) |
|  | Both 10% | 0.023 (0.166) | 0.000 |  | 83 (12) |
|  | Both 20% | -0.010 (0.194) | 0.013 | 32.7 | 37 (22) |
|  | Both 30% | -0.024 (0.308) | 0.179 | 19.6 | 8 (5) |
|  | Both 40% | -0.035 (0.391) | 0.338 | 9.6 | 4 (3) |
|  | Both 50% | -0.054 (0.445) | 0.496 | 5.9 | 3 (2) |
| Abandon | Kits 10% | 0.082 (0.138) | 0.000 |  | 97 (5) |
|  | Kits 20% | 0.070 (0.129) | 0.000 |  | 96 (5) |
|  | Kits 30% | 0.057 (0.119) | 0.000 |  | 95 (6) |
|  | Kits 40% | 0.044 (0.109) | 0.000 |  | 95 (6) |
|  | Kits 50% | 0.030 (0.100) | 0.000 |  | 94 (7) |
|  | Adults 10% | 0.052 (0.169) | 0.000 |  | 91 (9) |
|  | Adults 20% | 0.019 (0.193) | 0.000 |  | 79 (13) |
|  | Adults 30% | -0.002 (0.213) | 0.000 |  | 57 (18) |
|  | Adults 40% | -0.011 (0.244) | 0.000 |  | 30 (13) |
|  | Adults 50% | -0.017 (0.284) | 0.000 |  | 17 (7) |
|  | Both 10% | 0.041 (0.160) | 0.000 |  | 90 (9) |
|  | Both 20% | 0.003 (0.170) | 0.000 |  | 71 (14) |
|  | Both 30% | -0.011 (0.190) | 0.000 |  | 31 (11) |
|  | Both 40% | -0.018 (0.224) | 0.000 |  | 16 (5) |
|  | Both 50% | -0.022 (0.258) | 0.000 |  | 10 (3) |
| Isolated | Kits 10% | 0.056 (0.152) | 0.000 |  | 93 (8) |
|  | Kits 20% | 0.044 (0.144) | 0.000 |  | 92 (9) |
|  | Kits 30% | 0.031 (0.135) | 0.000 |  | 89 (10) |
|  | Kits 40% | 0.019 (0.126) | 0.000 |  | 86 (12) |
|  | Kits 50% | 0.007 (0.117) | 0.000 |  | 80 (15) |
|  | Adults 10% | 0.032 (0.182) | 0.000 |  | 85 (12) |
|  | Adults 20% | 0.000 (0.208) | 0.090 | 33.7 | 55 (28) |
|  | Adults 30% | -0.068 (0.268) | 0.966 | 22.7 | 1 (5) |
|  | Adults 40% | -0.159 (0.314) | 1.000 | 10.3 |  |
|  | Adults 50% | -0.262 (0.362) | 1.000 | 6.5 |  |
|  | Both 10% | 0.021 (0.171) | 0.000 |  | 83 (13) |
|  | Both 20% | -0.034 (0.211) | 0.654 | 30.0 | 11 (20) |
|  | Both 30% | -0.148 (0.258) | 1.000 | 11.3 |  |
|  | Both 40% | -0.280 (0.296) | 1.000 | 6.3 |  |
|  | Both 50% | -0.416 (0.338) | 1.000 | 4.4 |  |
