# Supplementary material for: Simulating Free-Roaming Cat Population Management Options in Open Demographic Environments
Source: PLoS One. 2014 Nov 26;9(11):e113553. doi: 10.1371/journal.pone.0113553 (PMC4245120; doi:10.1371/journal.pone.0113553)
Supplement: Table S9 — Full set of scenario results for the Sterilize management strategy applied to the Small Urban population. Column heading definitions are identical to those in Table S4. (DOCX) [file pone.0113553.s013.docx]

| **Scenario** | | **r_s_ (SD)** | **P(E)** | **T(E)** | **N_50_ (SD)** |
| --- | --- | --- | --- | --- | --- |
| Baseline | | 0.099 (0.143) | 0.000 |  | 98 (5) |
| Abandon/Dispersal | Kits 10% | 0.088 (0.134) | 0.000 |  | 97 (5) |
|  | Kits 20% | 0.077 (0.126) | 0.000 |  | 97 (5) |
|  | Kits 30% | 0.066 (0.118) | 0.000 |  | 97 (5) |
|  | Kits 40% | 0.053 (0.110) | 0.000 |  | 96 (5) |
|  | Kits 50% | 0.041 (0.102) | 0.000 |  | 95 (6) |
|  | Adults 10% | 0.057 (0.113) | 0.000 |  | 96 (5) |
|  | Adults 20% | 0.030 (0.094) | 0.000 |  | 94 (7) |
|  | Adults 30% | 0.014 (0.083) | 0.000 |  | 90 (8) |
|  | Adults 40% | 0.007 (0.077) | 0.000 |  | 86 (10) |
|  | Adults 50% | 0.003 (0.073) | 0.000 |  | 82 (10) |
|  | Both 10% | 0.049 (0.107) | 0.000 |  | 95 (6) |
|  | Both 20% | 0.019 (0.086) | 0.000 |  | 91 (8) |
|  | Both 30% | 0.006 (0.076) | 0.000 |  | 84 (10) |
|  | Both 40% | 0.001 (0.071) | 0.000 |  | 75 (11) |
|  | Both 50% | -0.001 (0.068) | 0.000 |  | 67 (10) |
| Dispersal | Kits 10% | 0.060 (0.147) | 0.000 |  | 94 (7) |
|  | Kits 20% | 0.048 (0.140) | 0.000 |  | 92 (8) |
|  | Kits 30% | 0.038 (0.133) | 0.000 |  | 91 (9) |
|  | Kits 40% | 0.027 (0.126) | 0.000 |  | 88 (10) |
|  | Kits 50% | 0.017 (0.119) | 0.000 |  | 84 (13) |
|  | Adults 10% | 0.027 (0.126) | 0.000 |  | 89 (11) |
|  | Adults 20% | 0.004 (0.117) | 0.000 |  | 70 (22) |
|  | Adults 30% | -0.008 (0.131) | 0.011 | 34.4 | 39 (22) |
|  | Adults 40% | -0.013 (0.145) | 0.016 | 29.8 | 25 (14) |
|  | Adults 50% | -0.016 (0.153) | 0.023 | 28.9 | 19 (9) |
|  | Both 10% | 0.020 (0.121) | 0.000 |  | 86 (12) |
|  | Both 20% | -0.003 (0.121) | 0.005 | 32.4 | 51 (23) |
|  | Both 30% | -0.012 (0.140) | 0.021 | 28.7 | 25 (13) |
|  | Both 40% | -0.016 (0.153) | 0.026 | 28.2 | 18 (7) |
|  | Both 50% | -0.017 (0.159) | 0.043 | 27.1 | 16 (6) |
| Abandon | Kits 10% | 0.083 (0.139) | 0.000 |  | 97 (5) |
|  | Kits 20% | 0.071 (0.130) | 0.000 |  | 96 (6) |
|  | Kits 30% | 0.059 (0.121) | 0.000 |  | 96 (6) |
|  | Kits 40% | 0.046 (0.113) | 0.000 |  | 95 (6) |
|  | Kits 50% | 0.033 (0.104) | 0.000 |  | 94 (7) |
|  | Adults 10% | 0.052 (0.116) | 0.000 |  | 95 (6) |
|  | Adults 20% | 0.024 (0.097) | 0.000 |  | 92 (8) |
|  | Adults 30% | 0.010 (0.086) | 0.000 |  | 87 (10) |
|  | Adults 40% | 0.003 (0.080) | 0.000 |  | 81 (11) |
|  | Adults 50% | 0.000 (0.076) | 0.000 |  | 74 (12) |
|  | Both 10% | 0.042 (0.110) | 0.000 |  | 95 (6) |
|  | Both 20% | 0.013 (0.089) | 0.000 |  | 88 (9) |
|  | Both 30% | 0.002 (0.078) | 0.000 |  | 76 (12) |
|  | Both 40% | -0.002 (0.073) | 0.000 |  | 64 (12) |
|  | Both 50% | -0.004 (0.070) | 0.000 |  | 54 (9) |
| Isolated | Kits 10% | 0.058 (0.153) | 0.000 |  | 93 (8) |
|  | Kits 20% | 0.045 (0.146) | 0.000 |  | 91 (9) |
|  | Kits 30% | 0.034 (0.137) | 0.001 | 45.5 | 90 (10) |
|  | Kits 40% | 0.021 (0.130) | 0.002 | 40.8 | 85 (13) |
|  | Kits 50% | 0.010 (0.124) | 0.022 | 35.9 | 76 (22) |
|  | Adults 10% | 0.024 (0.132) | 0.003 | 38.2 | 86 (14) |
|  | Adults 20% | -0.015 (0.138) | 0.427 | 30.8 | 34 (36) |
|  | Adults 30% | -0.084 (0.178) | 0.990 | 18.5 | 1 (2) |
|  | Adults 40% | -0.137 (0.220) | 1.000 | 11.8 |  |
|  | Adults 50% | -0.169 (0.261) | 1.000 | 9.7 |  |
|  | Both 10% | 0.016 (0.127) | 0.008 | 28.9 |  |
|  | Both 20% | -0.050 (0.158) | 0.873 | 24.9 |  |
|  | Both 30% | -0.119 (0.197) | 1.000 | 13.8 |  |
|  | Both 40% | -0.164 (0.252) | 1.000 | 10.0 |  |
|  | Both 50% | -0.185 (0.287) | 1.000 | 8.6 |  |
