# Supplementary material for: Simulating Free-Roaming Cat Population Management Options in Open Demographic Environments
Source: PLoS One. 2014 Nov 26;9(11):e113553. doi: 10.1371/journal.pone.0113553 (PMC4245120; doi:10.1371/journal.pone.0113553)
Supplement: Table S10 — Full set of scenario results for the Contracept-A management strategy applied to the Small Urban population. Column heading definitions are identical to those in Table S4. (DOCX) [file pone.0113553.s014.docx]

| **Scenario** | | **r_s_ (SD)** | **P(E)** | **T(E)** | **N_50_ (SD)** |
| --- | --- | --- | --- | --- | --- |
| Baseline | | 0.099 (0.143) | 0.000 |  | 98 (5) |
| Abandon/Dispersal | Kits 10% | 0.090 (0.137) | 0.000 |  | 98 (5) |
|  | Kits 20% | 0.083 (0.131) | 0.000 |  | 97 (5) |
|  | Kits 30% | 0.077 (0;127) | 0.000 |  | 97 (5) |
|  | Kits 40% | 0.069 (0.120) | 0.000 |  | 97 (5) |
|  | Kits 50% | 0.062 (0.116) | 0.000 |  | 97 (5) |
|  | Adults 10% | 0.066 (0.118) | 0.000 |  | 97 (5) |
|  | Adults 20% | 0.045 (0.104) | 0.000 |  | 95 (6) |
|  | Adults 30% | 0.030 (0.094) | 0.000 |  | 94 (6) |
|  | Adults 40% | 0.021 (0.087) | 0.000 |  | 93 (7) |
|  | Adults 50% | 0.014 (0.083) | 0.000 |  | 91 (8) |
|  | Both 10% | 0.061 (0.115) | 0.000 |  | 96 (5) |
|  | Both 20% | 0.036 (0.097) | 0.000 |  | 95 (6) |
|  | Both 30% | 0.021 (0.087) | 0.000 |  | 93 (7) |
|  | Both 40% | 0.011 (0.080) | 0.000 |  | 89 (8) |
|  | Both 50% | 0.005 (0.076) | 0.000 |  | 86 (9) |
| Dispersal | Kits 10% | 0.064 (0.150) | 0.000 |  | 94 (7) |
|  | Kits 20% | 0.057 (0.146) | 0.000 |  | 93 (8) |
|  | Kits 30% | 0.050 (0.141) | 0.000 |  | 93 (7) |
|  | Kits 40% | 0.043 (0.137) | 0.000 |  | 93 (8) |
|  | Kits 50% | 0.037 (0.133) | 0.000 |  | 91 (8) |
|  | Adults 10% | 0.037 (0.131) | 0.000 |  | 91 (9) |
|  | Adults 20% | 0.017 (0.118) | 0.000 |  | 85 (12) |
|  | Adults 30% | 0.006 (0.111) | 0.000 |  | 78 (16) |
|  | Adults 40% | -0.001 (0.110) | 0.000 |  | 65 (20) |
|  | Adults 50% | -0.006 (0.114) | 0.000 |  | 48 (21) |
|  | Both 10% | 0.032 (0.128) | 0.000 |  | 90 (9) |
|  | Both 20% | 0.011 (0.114) | 0.000 |  | 83 (13) |
|  | Both 30% | 0.000 (0.108) | 0.000 |  | 66 (19) |
|  | Both 40% | -0.007 (0.114) | 0.000 |  | 43 (19) |
|  | Both 50% | -0.012 (0.126) | 0.005 |  | 28 (12) |
| Abandon | Kits 10% | 0.086 (0.141) | 0.000 |  | 97 (5) |
|  | Kits 20% | 0.079 (0.135) | 0.000 |  | 97 (5) |
|  | Kits 30% | 0.071 (0.130) | 0.000 |  | 96 (5) |
|  | Kits 40% | 0.063 (0.125) | 0.000 |  | 96 (5) |
|  | Kits 50% | 0.057 (0;120) | 0.000 |  | 96 (6) |
|  | Adults 10% | 0.061 (0.123) | 0.000 |  | 96 (6) |
|  | Adults 20% | 0.040 (0.107) | 0.000 |  | 95 (6) |
|  | Adults 30% | 0.025 (0.098) | 0.000 |  | 95 (7) |
|  | Adults 40% | 0.015 (0.090) | 0.000 |  | 90 (8) |
|  | Adults 50% | 0.009 (0.086) | 0.000 |  | 88 (9) |
|  | Both 10% | 0.056 (0.119) | 0.000 |  | 95 (6) |
|  | Both 20% | 0.031 (0.101) | 0.000 |  | 93 (7) |
|  | Both 30% | 0.015 (0.091) | 0.000 |  | 90 (8) |
|  | Both 40% | 0.007 (0.083) | 0.000 |  | 86 (10) |
|  | Both 50% | 0.002 (0.079) | 0.000 |  | 80 (11) |
| Isolated | Kits 10% | 0.061 (0.156) | 0.000 |  | 94 (7) |
|  | Kits 20% | 0.053 (0.151) | 0.000 |  | 93 (8) |
|  | Kits 30% | 0.046 (0.146) | 0.000 |  | 92 (8) |
|  | Kits 40% | 0.040 (0.141) | 0.000 |  | 91 (9) |
|  | Kits 50% | 0.033 (0.138) | 0.000 |  | 90 (9) |
|  | Adults 10% | 0.034 (0.137) | 0.000 |  | 88 (11) |
|  | Adults 20% | 0.014 (0.124) | 0.000 |  | 83 (14) |
|  | Adults 30% | 0.002 (0.120) | 0.025 |  | 68 (24) |
|  | Adults 40% | -0.013 (0.130) | 0.247 |  | 37 (30) |
|  | Adults 50% | -0.036 (0.149) | 0.696 |  | 8 (16) |
|  | Both 10% | 0.029 (0.134) | 0.000 |  | 89 (10) |
|  | Both 20% | 0.008 (0.120) | 0.004 |  | 78 (17) |
|  | Both 30% | -0.011 (0.126) | 0.173 |  | 40 (29) |
|  | Both 40% | -0.041 (0.152) | 0.791 |  | 4 (11) |
|  | Both 50% | -0.067 (0.171) | 0.986 |  | 1 (1) |
