# Supplementary material for: Simulating Free-Roaming Cat Population Management Options in Open Demographic Environments
Source: PLoS One. 2014 Nov 26;9(11):e113553. doi: 10.1371/journal.pone.0113553 (PMC4245120; doi:10.1371/journal.pone.0113553)
Supplement: Table S11 — Full set of scenario results for the Contracept-B management strategy applied to the Small Urban population. Column heading definitions are identical to those in Table S4. (DOCX) [file pone.0113553.s015.docx]

| **Scenario** | | **r_s_ (SD)** | **P(E)** | **T(E)** | **N_50_ (SD)** |
| --- | --- | --- | --- | --- | --- |
| Baseline | | 0.099 (0.143) | 0.000 |  | 98 (5) |
| Abandon/Dispersal | Kits 10% | 0 093 (0.138) | 0.000 |  | 98 (5) |
|  | Kits 20% | 0.087 (0.134) | 0.000 |  | 97 (5) |
|  | Kits 30% | 0.081 (0.130) | 0.000 |  | 97 (5) |
|  | Kits 40% | 0.076 (0.126) | 0.000 |  | 97 (5) |
|  | Kits 50% | 0.071 (0.123) | 0.000 |  | 96 (5) |
|  | Adults 10% | 0.073 (0.123) | 0.000 |  | 97 (5) |
|  | Adults 20% | 0.054 (0.110) | 0.000 |  | 96 (5) |
|  | Adults 30% | 0.040 (0.099) | 0.000 |  | 95 (6) |
|  | Adults 40% | 0.030 (0.093) | 0.000 |  | 94 (6) |
|  | Adults 50% | 0.022 (0.088) | 0.000 |  | 93 (7) |
|  | Both 10% | 0.068 (0.120) | 0.000 |  | 97 (5) |
|  | Both 20% | 0.047 (0.105) | 0.000 |  | 96 (5) |
|  | Both 30% | 0.032 (0.095) | 0.000 |  | 94 (6) |
|  | Both 40% | 0.021 (0.087) | 0.000 |  | 93 (6) |
|  | Both 50% | 0.014 (0.082) | 0.000 |  | 91 (8) |
| Dispersal | Kits 10% | 0.065 (0.151) | 0.000 |  | 94 (7) |
|  | Kits 20% | 0.060 (0.148) | 0.000 |  | 93 (7) |
|  | Kits 30% | 0.055 (0.145) | 0.000 |  | 93 (7) |
|  | Kits 40% | 0.051 (0.142) | 0.000 |  | 93 (7) |
|  | Kits 50% | 0.046 (0.139) | 0.000 |  | 92 (8) |
|  | Adults 10% | 0.044 (0.136) | 0.000 |  | 92 (9) |
|  | Adults 20% | 0.025 (0.124) | 0.000 |  | 88 (10) |
|  | Adults 30% | 0.013 (0.115) | 0.000 |  | 84 (13) |
|  | Adults 40% | 0.006 (0.110) | 0.000 |  | 78 (15) |
|  | Adults 50% | 0.000 (0.108) | 0.000 |  | 68 (19) |
|  | Both 10% | 0.040 (0.134) | 0.000 |  | 91 (9) |
|  | Both 20% | 0.020 (0.120) | 0.000 |  | 87 (11) |
|  | Both 30% | 0.008 (0.111) | 0.000 |  | 81 (14) |
|  | Both 40% | 0.000 (0.108) | 0.000 |  | 69 (18) |
|  | Both 50% | -0.005 (0.109) | 0.002 |  | 50 (19) |
| Abandon | Kits 10% | 0.088 (0.143) | 0.000 |  | 97 (5) |
|  | Kits 20% | 0.082 (0.139) | 0.000 |  | 97 (5) |
|  | Kits 30% | 0.077 (0.134) | 0.000 |  | 96 (5) |
|  | Kits 40% | 0.071 (0.130) | 0.000 |  | 97 (5) |
|  | Kits 50% | 0.065 (0.127) | 0.000 |  | 96 (6) |
|  | Adults 10% | 0.067 (0.127) | 0.000 |  | 96 (5) |
|  | Adults 20% | 0.048 (0.113) | 0.000 |  | 96 (6) |
|  | Adults 30% | 0.034 (0.104) | 0.000 |  | 94 (7) |
|  | Adults 40% | 0.025 (0.096) | 0.000 |  | 92 (7) |
|  | Adults 50% | 0.017 (0.090) | 0.000 |  | 91 (8) |
|  | Both 10% | 0.063 (0.124) | 0.000 |  | 96 (6) |
|  | Both 20% | 0.041 (0.109) | 0.000 |  | 95 (6) |
|  | Both 30% | 0.026 (0.098) | 0.000 |  | 93 (7) |
|  | Both 40% | 0.016 (0.091) | 0.000 |  | 90 (8) |
|  | Both 50% | 0.009 (0.085) | 0.000 |  | 87 (9) |
| Isolated | Kits 10% | 0.064 (0.158) | 0.000 |  | 94 (8) |
|  | Kits 20% | 0.058 (0.154) | 0.000 |  | 93 (8) |
|  | Kits 30% | 0.052 (0.151) | 0.000 |  | 93 (8) |
|  | Kits 40% | 0.048 (0.147) | 0.000 |  | 92 (8) |
|  | Kits 50% | 0.042 (0.144) | 0.002 |  | 91 (8) |
|  | Adults 10% | 0.040 (0.142) | 0.000 |  | 91 (9) |
|  | Adults 20% | 0.022 (0.129) | 0.000 |  | 87 (11) |
|  | Adults 30% | 0.010 (0.121) | 0.000 |  | 81 (15) |
|  | Adults 40% | 0.001 (0.119) | 0.031 | 36.8 | 68 (24) |
|  | Adults 50% | -0.009 (0.123) | 0.158 | 34.4 | 45 (30) |
|  | Both 10% | 0.036 (0.138) | 0.000 |  | 90 (9) |
|  | Both 20% | 0.016 (0.124) | 0.000 |  | 84 (13) |
|  | Both 30% | 0.003 (0.118) | 0.017 | 34.0 | 72 (21) |
|  | Both 40% | -0.009 (0.122) | 0.125 | 34.9 | 45 (29) |
|  | Both 50% | -0.029 (0.141) | 0.536 | 33.5 | 13 (19) |
