# Supplementary material for: Simulating Free-Roaming Cat Population Management Options in Open Demographic Environments
Source: PLoS One. 2014 Nov 26;9(11):e113553. doi: 10.1371/journal.pone.0113553 (PMC4245120; doi:10.1371/journal.pone.0113553)
Supplement: Table S12 — Full set of scenario results for the Removal management strategy applied to the Rural population. Column heading definitions are identical to those in Table S4. (DOCX) [file pone.0113553.s016.docx]

| **Scenario** | | **r_s_ (SD)** | **P(E)** | **T(E)** | **N_50_ (SD)** |
| --- | --- | --- | --- | --- | --- |
| Baseline | | 0.027 (0.194) | 0.074 | 30.4 | 19 (7) |
| Isolated | Kits 10% | 0.019 (0.186) | 0.095 | 28.8 | 18 (7) |
|  | Kits 20% | 0.010 (0.182) | 0.211 | 28.4 | 15 (9) |
|  | Kits 30% | 0.000 (0.178) | 0.371 | 27.6 | 11 (9) |
|  | Kits 40% | -0.011 (0.177) | 0.569 | 26.6 | 7 (9) |
|  | Kits 50% | -0.027 (0.178) | 0.817 | 24.3 | 2 (6) |
|  | Adults 10% | -0.006 (0.232) | 0.616 | 25.1 | 6 (8) |
|  | Adults 20% | -0.065 (0.280) | 0.992 | 13.5 | 1 (1) |
|  | Adults 30% | -0.139 (0.319) | 1.000 | 7.1 |  |
|  | Adults 40% | -0.224 (0.347) | 1.000 | 4.7 |  |
|  | Adults 50% | -0.312 (0.407) | 1.000 | 3.4 |  |
|  | Both 10% | -0.018 (0.229) | 0.749 | 23.5 | 3 (6) |
|  | Both 20% | -0.101 (0.265) | 1.000 | 9.7 |  |
|  | Both 30% | -0.197 (0.292) | 1.000 | 5.3 |  |
|  | Both 40% | -0.302 (0.324) | 1.000 | 3.7 |  |
|  | Both 50% | -0.414 (0.375) | 1.000 | 2.8 |  |
