# Supplementary material for: Simulating Free-Roaming Cat Population Management Options in Open Demographic Environments
Source: PLoS One. 2014 Nov 26;9(11):e113553. doi: 10.1371/journal.pone.0113553 (PMC4245120; doi:10.1371/journal.pone.0113553)
Supplement: Table S13 — Full set of scenario results for the Sterilize management strategy applied to the Rural population. Column heading definitions are identical to those in Table S4. (DOCX) [file pone.0113553.s017.docx]

| **Scenario** | | **r_s_ (SD)** | **P(E)** | **T(E)** | **N_50_ (SD)** |
| --- | --- | --- | --- | --- | --- |
| Baseline | | 0.027 (0.194) | 0.074 | 30.4 | 19 (7) |
| Isolated | Kits 10% | 0.019 (0.189) | 0.156 | 27.9 | 17 (8) |
|  | Kits 20% | 0.009 (0.185) | 0.330 | 29.1 | 13 (10) |
|  | Kits 30% | -0.003 (0.183) | 0.545 | 28.1 | 8 (9) |
|  | Kits 40% | -0.017 (0.184) | 0.779 | 25.2 | 3 (7) |
|  | Kits 50% | -0.032 (0.186) | 0.934 | 22.7 | 1 (3) |
|  | Adults 10% | -0.021 (0.185) | 0.829 | 22.8 | 3 (6) |
|  | Adults 20% | -0.070 (0.191) | 0.997 | 13.4 | 1 (1) |
|  | Adults 30% | -0.100 (0.180) | 1.000 | 9.0 |  |
|  | Adults 40% | -0.130 (0.199) | 1.000 | 7.8 |  |
|  | Adults 50% | -0.132 (0.184) | 1.000 | 7.0 |  |
|  | Both 10% | -0.030 (0.186) | 1.000 | 21.4 |  |
|  | Both 20% | -0.083 (0.188) | 1.000 | 11.7 |  |
|  | Both 30% | -0.118 (0.196) | 1.000 | 8.5 |  |
|  | Both 40% | -0.139 (0.196) | 1.000 | 7.4 |  |
|  | Both 50% | -0.148 (0.197) | 1.000 | 7.0 |  |
