# Supplementary material for: Simulating Free-Roaming Cat Population Management Options in Open Demographic Environments
Source: PLoS One. 2014 Nov 26;9(11):e113553. doi: 10.1371/journal.pone.0113553 (PMC4245120; doi:10.1371/journal.pone.0113553)
Supplement: Table S14 — Full set of scenario results for the Contracept-A management strategy applied to the Rural population. Column heading definitions are identical to those in Table S4. (DOCX) [file pone.0113553.s018.docx]

| **Scenario** | | **r_s_ (SD)** | **P(E)** | **T(E)** | **N_50_ (SD)** |
| --- | --- | --- | --- | --- | --- |
| Baseline | | 0.027 (0.194) | 0.074 | 30.4 | 19 (7) |
| Isolated | Kits 10% | 0.033 (0.188) | 0.041 | 28.2 | 20 (6) |
|  | Kits 20% | 0.022 (0.190) | 0.076 | 30.2 | 19 (7) |
|  | Kits 30% | 0.012 (0.184) | 0.187 | 30.2 | 15 (8) |
|  | Kits 40% | 0.008 (0.182) | 0.223 | 29.5 | 14 (9) |
|  | Kits 50% | 0.002 (0.182) | 0.294 | 28.2 | 12 (9) |
|  | Adults 10% | -0.005 (0.165) | 0.852 | 20.9 | 3 (7) |
|  | Adults 20% | -0.025 (0.185) | 0.832 | 23.1 | 2 (6) |
|  | Adults 30% | -0.046 (0.190) | 0.967 | 18.5 | 1 (3) |
|  | Adults 40% | -0.061 (0.190) | 0.991 | 15.5 | 1 (0) |
|  | Adults 50% | -0.082 (0.194) | 0.999 | 12.3 |  |
|  | Both 10% | -0.005 (0.183) | 0.530 | 26.9 | 8 (9) |
|  | Both 20% | -0.033 (0.187) | 0.904 | 21.5 | 1 (4) |
|  | Both 30% | -0.059 (0.189) | 0.996 | 16.3 | 1 (1) |
|  | Both 40% | -0.075 (0.192) | 0.999 | 13.1 |  |
|  | Both 50% | -0.092 (0.194) | 1.000 | 11.0 |  |
