# Supplementary material for: Simulating Free-Roaming Cat Population Management Options in Open Demographic Environments
Source: PLoS One. 2014 Nov 26;9(11):e113553. doi: 10.1371/journal.pone.0113553 (PMC4245120; doi:10.1371/journal.pone.0113553)
Supplement: Table S15 — Full set of scenario results for the Contracept-B management strategy applied to the Rural population. Column heading definitions are identical to those in Table S4. (DOCX) [file pone.0113553.s019.docx]

| **Scenario** | | **r_s_ (SD)** | **P(E)** | **T(E)** | **N_50_ (SD)** |
| --- | --- | --- | --- | --- | --- |
| Baseline | | 0.027 (0.194) | 0.074 | 30.4 | 19 (7) |
| Isolated | Kits 10% | 0.023 (0.191) | 0.087 | 28.8 | 18 (7) |
|  | Kits 20% | 0.020 (0.188) | 0.110 | 31.4 | 18 (7) |
|  | Kits 30% | 0.016 (0.187) | 0.135 | 27.9 | 17 (8) |
|  | Kits 40% | 0.013 (0.185) | 0.187 | 29.3 | 16 (8) |
|  | Kits 50% | 0.010 (0.183) | 0.196 | 28.1 | 15 (9) |
|  | Adults 10% | 0.007 (0.184) | 0.304 | 27.1 | 13 (9) |
|  | Adults 20% | -0.012 (0.183) | 0.649 | 25.6 | 5 (8) |
|  | Adults 30% | -0.030 (0.185) | 0.878 | 22.0 | 2 (5) |
|  | Adults 40% | -0.043 (0.188) | 0.955 | 19.1 | 1 (3) |
|  | Adults 50% | -0.058 (0.189) | 0.989 | 16.1 |  |
|  | Both 10% | 0.003 (0.182) | 0.382 | 27.7 | 11 (10) |
|  | Both 20% | -0.019 (0.183) | 0.743 | 24.4 | 4 (7) |
|  | Both 30% | -0.039 (0.186) | 0.938 | 20.4 | 1 (3) |
|  | Both 40% | -0.056 (0.190) | 0.990 | 16.5 |  |
|  | Both 50% | -0.070 (0.190) | 0.999 | 27.8 |  |
