# Supplementary material for: Simulating Free-Roaming Cat Population Management Options in Open Demographic Environments
Source: PLoS One. 2014 Nov 26;9(11):e113553. doi: 10.1371/journal.pone.0113553 (PMC4245120; doi:10.1371/journal.pone.0113553)
Supplement: File S1 — Main supporting information file. This file includes additional materials and methods (model structure and input data), additional results (elasticity analysis), and additional references. (DOCX) [file pone.0113553.s020.docx]

**Supporting Information**

**Supporting Methods**

Baseline Input Parameters for Demographic Simulation Models

Data for use as input to our simulation models were obtained from the peer-reviewed literature where available and appropriate. We created a database of literature and, subsequently, a matrix of demographic values, corresponding to specific *Vortex* input fields where available (this matrix is available on the ACC&D website). Where specific data were not available in the scientific literature, we used expert judgment to derive input parameter values through a process of consensus. More detailed information on the underlying structure, algorithms and approach used in *Vortex* is available in the complete software User’s Manual [1].

Timestep for all simulations: The timestep for this type of demographic analysis defines the interval over which demographic rates are applied to individuals within the population, and over which summary population statistics are calculated. Because free-roaming cats tend to have a more seasonal pattern of reproduction, and early onset of reproductive activity, we used a timestep for our simulations of six months. On a calendar basis, these timesteps would roughly correspond to a “spring/summer” timestep featuring high reproductive rates, and an “autumn/winter” timestep with correspondingly lower reproductive rates. All reproductive and survival probabilities are therefore calculated on the basis of this timestep.

Metapopulation structure: For our Large Urban and Small Urban scenarios, we employed a simplified metapopulation structure. This means we set up two subpopulations that together comprised the metapopulation: the focal population to which all of our treatment scenarios were directed, and the “neighborhood” to which that focal population was demographically connected through dispersal of individual animals. This configuration required us to specify demographic input parameters for the neighborhood population as well, even if we are not interested in tracking the long-term dynamics of that population. In all of our Urban scenarios, we assumed that the neighborhood population has the same basic demographic structure as our focal population, in other words, equivalent rates of reproduction and survival. The only difference is that the neighborhood population is four times larger than the focal population in both initial population size and carrying capacity (see below).

Dispersal in our Urban metapopulation scenarios must be characterized in some detail. Although many studies document the presence of immigration and emigration among local cat populations [e.g., 2 – 5], the specific demographic characteristics of those dispersing individuals are not similarly available from the literature. Therefore, we assumed that dispersing individuals are between 6 and 24 months of age, with males making up approximately 75% of the dispersing individuals. We assumed a rate of dispersal between focal and neighborhood populations of 2% per timestep. This means that, on average, a population of 100 individuals at a given point in time will have two individuals of the appropriate age-sex class randomly chosen to disperse to the other population. Although these rates of dispersal between focal and neighborhood populations are the same, the 4-fold larger size of the surrounding neighborhood population means that the number of individuals entering the focal population was considerably larger than the number that leaving that population. Finally, we also assumed there is a cost to dispersal across human-dominated urban landscapes, expressed as an additional 25% mortality of those individuals designated as dispersers in any given timestep.

Breeding system: Free-roaming cats display a classic polygynandrous breeding system, where both males and females may mate with multiple partners. Individual litters with multiple paternity have been observed, but this is not important from a population demographic standpoint as we are not explicitly including population-level genetic considerations in our analyses, and we assumed that the size of litters sired by multiple males will be the same as those sired by a single male.

Age of first reproduction: Free-roaming cats become reproductive at 6-12 months of age (median age 10.5 months, interquartile range 8 – 12 months: [6]). For our purposes, we set the age of first reproduction at 6 months, meaning that an individual reaching 6 months of age will have an opportunity to breed once before aging to the next timestep (i.e., before becoming 12 months old).

Maximum age of reproduction: In its simplest form, *Vortex* assumes that animals can reproduce (at the normal rate) throughout their adult life. Under this assumption, individuals can reproduce until they die, meaning that there is no reproductive senescence in our model. Free-roaming cats are clearly capable of breeding successfully for many years if they escape mortality from natural or anthropogenic causes. There is considerable variance around estimates of typical free-roaming cat longevity. Many sources suggest a typical lifespan of approximately 3 – 5 years of age [7, 8]. We assumed for our models a maximum possible age of 6.5 years (i.e., 13 timesteps). In other words, an animal that reaches 6.5 years of age – 13 time steps – is allowed to go through one final breeding cycle before being removed from the population through additional mortality. Note that the probability of a newborn kitten actually reaching this age is no more than about 10%. This maximum age may be an optimistic estimate, but we evaluate reproductive management options while effectively holding this longevity parameter value constant across all scenarios.

Kitten production: For all models discussed here, we defined reproduction as the successful live birth of a litter of kittens, before weaning has taken place. An extensive dataset [6] showed litter production at an average rate of 1.4 litters per female per year, demonstrating the ability of adult females to produce more than one litter in a year. Furthermore, their data suggested a strong seasonality to litter production. We specified in *Vortex* that an adult female will produce no more than one litter in any one timestep. Assuming a rough 2:1 ratio of litters produced in the “spring/summer” timestep compared to “autumn/winter”, and given the overall mean litter production per year, we then assumed that on average 92% of free-roaming adult females produced a single litter in the more favorable timestep, and 48% produced a litter in the less favorable timestep (Figure S1). We recognized that, in theory, a given female could actually produce both litters in the same 6-month interval, thereby failing to reproduce in the next interval. The structure of our model did not allow for this event, but we were confident that the overall demographic result of our model structure is very similar to that which may occur in nature.

We allowed the breeding rate to vary randomly from one breeding cycle to the next to account for random environmental factors influencing mean rates. This source of variability is often referred to as environmental stochasticity, and is defined operationally as a standard deviation applied to the mean rates at each timestep. As in the case of the mean rates, we specified a seasonal form of environmental variability, with a standard deviation in the “spring/summer” timestep of 3% and 12% in the “autumn/winter” timestep. We assumed that reproduction in the lower-productivity season will be more highly variable than the more optimal season.

Data on litter size [6] indicated a median of 3 kittens/litter, with a range of 1 to 6. Note that this median value does not include stillbirths and/or aborted fetuses, but instead specifies live births. We agreed on a mean distribution of 3.47 kittens/litter, with the specific distribution as described in Table S1. Across all litters born in a given timestep, we assume a 50:50 sex ratio.

Density dependence in reproduction: The inclusion of density dependence is an important consideration in demographic modeling efforts such as this. We chose a strategy where high density does not reduce the proportion of females that produce litters, but instead reduces the survivorship of those kittens after they are born. For more details, see the discussion on mortality rates below.

Male breeding rates: In some species, a proportion of adult males may be socially restricted from breeding despite being physiologically capable. This can be modeled in *Vortex* by specifying a portion of the total pool of adult males that may be considered “available” for breeding each timestep. We do not have detailed data on this parameter, so we assumed that all adult males are available for breeding each timestep. This is most probably a reasonable assumption, as population dynamics of polygynous species such as the domestic cat are very rarely male-limited. In the model structure employed by *Vortex*, males in a polygynous breeding system will not be limiting, as individuals may be used multiple times for mating with the group of available females.

Mortality rates: *Vortex* defines mortality as the annual rate of age-specific death from timestep *x* to *x* + 1; in the language of life-table analysis, this is equivalent to *q*(*x*). We assigned a mean value of 75% mortality for kittens from live birth to six months of age [6], with an assigned standard deviation around that mean due to environmental variability of 10%. While there are other estimates of lower kitten mortality (e.g., [4]), we believed that the study cited above provides a robust estimate of this parameter. If an adult female produces two litters in a given year, we assumed equal mortality across both litters, in accord with a previous study [6] that observed no significant influence of litter order on mortality. We recognized, however, that high-density cat populations will likely experience higher levels of kitten mortality as food and other resources become more limiting. We assumed that kitten mortality would increase to a value of 90% when the population abundance approached the local habitat carrying capacity, *K* (see below for more information on this parameter). The specific functional form of this relationship is:

where *S*_0_ is the survival at low density, *S_K_* is the survival rate at high density, *N* is population size, and *K* is carrying capacity (Figure S2). If specific scenarios called for higher kitten mortality rates, the same basic density-dependent relationship shown in the equation above continued to hold but the curve was shifted upward by an amount specified for that particular scenario.

Estimates of annual adult survival rates vary widely across the literature, from just under 0.9 in an urban population [9] to 0.55 – 0.78 in a rural population [5]. It may not be surprising to see higher adult survival in urban areas where supplemental feeding may be frequent, and overall resource availability may be enhanced. An urban annual survival rate around 90% appears commonly in the literature, so we agreed to set this as our baseline rate for Large and Small Urban populations. This equated to a survival rate of 0.948 per 6-month timestep, or a mortality rate (in the language of *Vortex*) of 0.052 per timestep.

In keeping with earlier studies (e.g., [10]), we assumed that both kitten and adult mortality rates would be higher in the Rural population type through greater competition for scarce resources, higher predator density, etc. Specifically, we set kitten (0 – 6 month) mortality at 80% with a similar density-dependent relationship featuring 95% mortality at high population density. Additionally, we increased adult mortality from 0.052 per timestep to 0.072 (equivalent to 13.9% annual mortality). This may be a conservative estimate of adult mortality in rural landscapes, but served as a useful baseline upon which reproductive management options were compared across population types.

We did not explore the impact of geographic location of free-roaming cat populations and the resulting impact on demography. For example, we considered that populations in higher latitudes would potentially experience greater seasonal variation in reproduction and/or mortality. Future versions of the model structure described here could be adapted to include this factor.

Catastrophes: Catastrophes are unusual environmental events that are outside the bounds of normal environmental variation affecting reproduction and/or survival. Natural catastrophes can be tornadoes, floods, droughts, disease, or similar events. These events are modeled in *Vortex* by assigning an annual probability of occurrence and a pair of severity factors describing their impact on mortality (across all age-sex classes) and on the proportion of females successfully breeding in a given year.

We agreed to exclude catastrophic events from our current set of models for purposes of simplicity. The mechanisms and impacts of such events on free-roaming cat populations, particularly those inhabiting highly modified environments in urban landscapes, are not well understood at this point. Future modeling efforts may be conducted to explicitly investigate the sensitivity of model outcome to the addition of catastrophes.

Inbreeding depression: *Vortex* provides the option to model the detrimental effects of inbreeding, most directly through reduced survival of offspring through their first 6-12 months. Detailed data on the presence and intensity of inbreeding and its impacts on free-roaming cat populations are not available. The impact of past inbreeding history, and the social structure of free-roaming cat populations, makes an estimate of inbreeding depression extent and severity highly speculative at best. Observed morphological diversity among free-roaming cats suggests that they possess at least moderate underlying genetic heterogeneity. Therefore, we excluded inbreeding depression as an additional factor in our models.

Initial population size and carrying capacity: All models were initialized with a specific population abundance and an expression of the ecological carrying capacity of the habitat. The ecological carrying capacity, *K*, for a given habitat patch defined an upper limit for the population size, above which additional mortality was imposed randomly across all age classes in order to return the population to the value set for *K*. In all models constructed for this report, we assumed that both the focal and (where applicable) neighborhood populations begin the simulations with an initial abundance equivalent to the carrying capacity for that habitat. This is a reasonable starting condition for analysis of reproductive management of free-roaming cat populations, as management issues will become highest priority when cat populations reach their maximum density and begin to generate higher levels of disease transmission risk, nuisance behaviors, and the like.

Carrying capacity values for each population type were estimated by assigning a density to each type, and extrapolating that value throughout its full spatial extent of 50Ha = 0.5km^2^. This method assumed, of course, that the assigned density represented something close to a maximum value that could most accurately reflect a meaningful carrying capacity estimate. The density values used for this analysis were derived from a summary [11] citing multiple original data sources. Initial population abundance and carrying capacity estimates used in our analyses are shown in Table S2.

Litter abandonment through “supplementation”: Selected scenarios included the abandonment of litters from owned-cat households. We implemented this through the Supplementation module in *Vortex* that allows the user to add a specified number of individuals of a given age/sex class at a user-defined frequency. We assumed that one “litter equivalent” is added to the focal population each 6-month timestep, composed of two males and two females all six months of age. We used the concept of “litter equivalent” to represent one abandoned litter with 100% survival to the next timestep, or two abandoned litters with 50% survival (perhaps abandoned at a younger age and subject to higher mortality before the next timestep), etc. to create a total of four animals that were added to the focal population.

Iterations and years of projection: Our stochastic simulation model features random variation in demographic parameters, yielding different results for different runs. Therefore, we generated multiple replicates to assess overall simulated population behavior. All scenarios were simulated 1000 times, with each projection extending to 100 timesteps (equivalent to 50 years).

Model Structures to Simulate Population Management Options

Remove: To simulate the set of proposed population management scenarios, we employed the concept of Individual State Variables (ISVs) within *Vortex*. These variables were assigned numerical values that undergo transitions through time depending on the nature of the scenario under consideration. In our Remove scenarios, we create the ISV *Trapped* that defined whether an individual had been trapped and therefore subjected to removal from the population. All individuals in the initial population were assigned *Trapped* = 0, meaning they had not been trapped prior to the onset of the simulation (in the language of *Vortex*, Initialization function = 0 for these individuals). When trapping kittens, we specified that a given percentage of individuals born in a given timestep were “tagged” for trapping according to defined probabilities. For these individuals, ISV *Trapped* transitions to 1 and each of these individuals were then assigned a 100% “mortality” probability. We recognized, of course, that these individuals do not literally die but were instead removed from the population permanently. Those kittens that were not trapped were subject to the baseline density-dependent mortality rate discussed above. We structured the trapping and removing of adults in a very similar manner, with transitions for each individual governed by defined probabilities that simulated the desired proportion of the population to be trapped.

We tested removal rates in the focal population of 10%, 20%, 30%, 40%, and 50% of the standing population of kittens, adults, or both groups combined, calculated at each 6-month timestep. Males and females were assumed to be trapped with equal probability.

Sterilize: We created the ISV *ReproState* that defined the reproductive state of any individual in the focal population. *ReproState* = 0 means an individual was reproductively active, while *ReproState* = 1 means they were reproductively inactive, i.e., sterilized. Within any given sterilization scenario, all individuals began the simulation as active (in the language of *Vortex*, Initialization function = 0), and all individuals born during the simulation started their lives reproductively active (i.e., Birth function = 0). An individual would “transition” from the active to the inactive state if trapped according to a specified probability. For example, if adults were targeted for sterilization (Age = A > 1 timestep), and the goal was to trap 10% of the total adult population, the *Vortex* transition function for each qualifying individual would flip an individual from ISV = 0 to ISV = 1 if a random number drawn for that individual was less than or equal to the probability of being trapped, in this case, 0.1. Importantly, in this and other scenarios described below, we simulated a system where trapped individuals were treated only if they had not been treated previously (i.e., *ReproState* = 0). Logically, then, we assumed over the time period of the simulation that a larger proportion of the total population would have to be trapped to achieve a given target treatment rate. The process of trapping was not explicitly modeled here; only the proportion of individuals treated was explicitly considered.

We defined detailed transition functions to specify whether kittens, adults, or both were targeted for trapping and sterilization. To simulate trapping of kittens, we identified individuals as Age = 1, corresponding to those individuals aged 6 months. While these individuals may be older than what is trapped in reality, the sequence of events in *Vortex* allowed the user to remove 6-month-old individuals before they had their first opportunity to breed. This was then largely equivalent to removing younger individuals as is done more realistically in the field.

We also specified that only individuals in our focal population were trapped and sterilized. Once sterilized, these individuals were allowed to disperse to the neighborhood population and remain reproductively inactive. Similarly, individuals in the neighborhood population could randomly disperse to the focal population and therefore be susceptible to trapping and sterilization.

Once trapped and treated, a sterilized individual’s probability of breeding was taken to be 0. A reproductively active female would breed at the baseline rate specified in the previous section. If a male was trapped and sterilized, it is considered no longer a member of the “pool” of available mates, so the total available pool was adjusted accordingly proportional to the total number of sterilized males.

We tested sterilization rates equal to 10%, 20%, 30%, 40%, and 50% of the untreated population of kittens, adults, or both groups combined, calculated at each 6-month timestep. Males and females were assumed to be trapped with equal probability. These rates were applied each timestep of the simulation, with another State Variable created to tally the total proportion of individuals within the focal population that were treated.

Contracept-A: We defined Individual State Variable *ReproState* in a manner that was similar to that for our Sterilize scenarios. However, built into this state variable was a type of “clock” that kept track of how many timesteps had passed since an individual transitioned from reproductively active to contracepted. This was necessary since the Contracept-A option featured full sterilization of an individual but for only three years (six timesteps). Once the “clock” reached three years for the contracepted state, the individual’s *ReproState* would reset from 1 (inactive) to 0 (active). When a treated individual reverted to a reproductively active state, they were then subject to trapping and retreatment as before. As with the Sterilize, all individuals within the focal population began the simulation as reproductively active, and all kittens were born active as well.

We also specified that only individuals in our focal population were trapped and contracepted. Once treated, these individuals could disperse to the neighborhood population and remain reproductively inactive. Similarly, individuals in the neighborhood population were allowed to randomly disperse to the focal population and therefore be susceptible to trapping and contraception.

We tested contraception rates equal to 10%, 20%, 30%, 40%, and 50% of the untreated population of kittens, adults, or both groups combined, calculated at each 6-month timestep. Males and females were assumed to be trapped with equal probability.

Contracept-B: We defined this management option very similarly to Contracept-A, with the same Individual State Variable *ReproState* and a “clock” that tallied how many timesteps had passed since an individual transitioned from reproductively active to contracepted. In this case, the “clock” was a separate ISV, *StepsSinceTreated*, which started at 0 for each individual and incremented upwards after an animal was treated (*ReproState* > 0). As before, all individuals within the focal population began the simulation as reproductively active, and all kittens were born active as well.

This final treatment option added some complexity to model structure in that an individual female’s return to fertility was a probabilistic function of time since treatment. We used data from a recent study [12] to derive a functional relationship between proportional return of fertility for females treated with the GnRH vaccine GonaCon^TM^ and the time since treatment (Figure S3). A linear relationship described the data very effectively (ANOVA: *F* = 238.12, *P* < 0.001) and we therefore adopted it as our approach to simulate a probabilistic transition to fertility. We constrained the linear regression to pass through the origin, thereby assuming that proportional fertility for treated females would be equal to or very nearly equal to 0.0 immediately after contraception. Given this assumption, the mathematical form of this relationship was simply

Prop. Fertility = 0.0734*(T)

where T was the number of timesteps since treatment. When a treated individual reverted to a reproductively active state, they were then subject to trapping and retreatment as before.

We also specified that only individuals in our focal population were trapped and contracepted. Once treated, these individuals could disperse to the neighborhood population and remain reproductively inactive. Similarly, individuals in the neighborhood population were allowed to randomly disperse to the focal population and therefore be susceptible to trapping and contraception.

We tested contraception rates equal to 10%, 20%, 30%, 40%, and 50% of the untreated population of kittens, adults, or both groups combined, calculated at each 6-month timestep. Males and females were assumed to be trapped with equal probability.

*Vortex* Syntax for Individual State Variable Descriptions and Demographic Impacts

Below is a listing of the specific equations describing the set of Individual State Variables (ISVs) used to simulate the four population management options, and the equations used to describe their impacts on free-roaming cat reproduction or mortality. The specific elements of Individual State Variable characterization are:

Initialization Function (Init fn): The value of that ISV assigned to all individuals making up the initial population at the beginning of the simulation.

Birth Function (Birth fn): The value of that ISV assigned to all individuals born into the population at each timestep.

Transition Function (Trans fn): The mathematical formulation describing the change in ISV value from one timestep to the next.

Variables used in the ISV functions:

*A* Age

*P* Population number, a numerical identifier for each subpopulation in a metapopulation (used to restrict treatment to focal population (P = 1))

*RAND* Uniform random number in the interval (0 – 1)

*x*  Threshold value for trapping/treating success

Management Strategy: Removal

| ISV | Label | Init fn | Birth fn | Trans fn |
| --- | --- | --- | --- | --- |
| IS1 | TrappedKits | 0 | =IS1+((P=1)*(RAND<x)) | =IS1 |
| IS2 | TrappedAdults | 0 | 0 | =IS2+((P=1)*(RAND<x)) |

Kitten mortality rate = (Trapped)(100%) + (NotTrapped)(Normal density-dependent mortality)

=((IS1=1)*100)+((IS1=0)*(100-(25-((25-10)*((N/K)^6)))

Adult mortality rate = (Trapped)(100%) + (NotTrapped)(Normal mortality)

=((IS2=1)*100)+((IS2=0)*5.2)

Management Strategy: Sterilize

| ISV | Label | Init fn | Birth fn | Trans fn |
| --- | --- | --- | --- | --- |
| IS1 | ReproStateKit | 0 | 0 | =IS1+((A=1)*(IS1=0)*(P=1)*(RAND<x)) |
| IS1 | ReproStateAd | 0 | 0 | =IS1+((A>1)*(IS1=0)*(P=1)*(RAND<x)) |

Female breeding rate = (ReproState=0)(Normal breeding rate)

=(IS1=0)*(48+(44*(Y%2=1))[Modulus (Y%2=1) controls seasonal rates]

Management Strategy: Contracept-A

| ISV | Label | Init fn | Birth fn | Trans fn |
| --- | --- | --- | --- | --- |
| IS1 | ReproStateKit | 0 | 0 | =((IS1=0)*(A=1)*(P=1)*(6*(RAND<x)))+((IS1>0)*(IS1-1)) |
| IS1 | ReproStateAd | 0 | 0 | =((IS1=0)*(A>1)*(P=1)*(6*(RAND<x)))+((IS1>0)*(IS1-1)) |

Female breeding rate = (ReproState=0)(Normal breeding rate)

=(IS1=0)*(48+(44*(Y%2=1))[Modulus (Y%2=1) controls seasonal rates]

Management Strategy: Contracept-B

| ISV | Label | Init fn | Birth fn | Trans fn |
| --- | --- | --- | --- | --- |
| IS1 | ReproStateKit | 0 | 0 | =[(IS1=0)*(A=1)*(P=1)*(RAND<x)]  +[(IS1=1)*(RAND>((0.0734)*IS2))] |
| IS1 | ReproStateAd | 0 | 0 | =[(IS1=0)*(A>1)*(P=1)*(RAND<x)]  +[(IS1=1)*(RAND>((0.0734)*IS2))] |
| IS2 | StepsSinceTreated | 0 | 0 | =(IS1>0)*(IS2+1) |

Female breeding rate = (ReproState=0)(Normal breeding rate)

=(IS1=0)*(48+(44*(Y%2=1))[Modulus (Y%2=1) controls seasonal rates]

**Supporting Results**

Elasticity Analysis

One of the primary goals of this analysis was to determine the major demographic factors – typically focused on age-specific survival or fecundity – driving population growth in our simulated free-roaming cat populations. This can give us valuable insight into the types of management actions that may yield the greatest benefit on the basis of their action on sensitive demographic parameters. We calculated a value known as the sensitivity of a given demographic rate [13], defined as the proportional change in population growth rate following a small change in the demographic rate of interest. Elasticity is further defined as a proportional form of sensitivity, which allows for more direct comparison of sensitivity values across demographic rates that may be on different scales, such as survival (constrained to be ≤ 1.0) and fecundity (usually >1.0) [14].

To calculate elasticity values for our baseline model, we created a simple, female-only stage-based demographic transition matrix using the simulation modeling package *RAMAS Metapop* [15]. This matrix described the reproductive rates (otherwise known as fecundity) and survival rates for kittens (just before they turn 6 months old) and adults (just before they turn 12 months old) on the familiar 6-month timestep in our Large Urban population type. This matrix was in a form shown in Table S2, with fecundity rates (expressed as female offspring surviving to six months of age produced per female) listed in the top row and 6-month survival rates in the bottom row. The fecundity values combined the probability of breeding (itself an average of primary season breeding rate = 0.92 and secondary season breeding rate = 0.48), average litter size = 3.47, survival to six months of age = 0.25, and proportion of female offspring = 0.5. The values in the two rows are identical because we have defined rates of reproduction and survival for all individuals >6 months of age to be the same.

We then calculated the growth rate defined by this baseline matrix. With this information in hand, we calculated the elasticity of each matrix element according to the equation

where λ*_x_*_+0.05_ is the deterministic growth rate (λ = e*^r^*) calculated from the above matrix with parameter *x* increased or decreased by 5%. The difference in the two population growth rates was then divided by the growth rate from the baseline unmodified matrix λ*_x_*, multiplied by the total modification. A larger elasticity value for a given demographic parameter indicates a greater level of sensitivity within the demographic model to change in that parameter. In accordance with this type of analytical elasticity analysis, the simulated population was initiated with a stable stage structure.

The average elasticity value for survival parameters was 0.3714 while the average value for fecundity parameters was 0.1198 (Figure S4), indicating that the baseline free-roaming cat model developed here was more sensitive to changes in age-specific survival than fecundity. In particular, as a result of the demographic values used for our baseline models, adult survival showed the greatest level of sensitivity. This result may seem counter-intuitive, especially when considered on the basis of comparative value of a younger vs. older individual to the long-term growth of a population. However, the low baseline kitten survival rate already in place means that a given unit change to that rate, or a similar change to kitten production, will not yield a large increase in the number of adults that emerge from that youngest age class. Moreover, kittens reside in that age class for just one timestep, after which time they transition into adults. Increasing survival in that class is therefore not compounded over a long time period. On the other hand, increasing adult survival across years compounds over multiple timesteps, yielding much larger increases in the number of adults and, by extension, larger increases in the number of offspring. This type of logic in sensitivity analysis was used in a classic study of marine turtles [14] to argue for management aimed at increasing adult survival through the use of turtle excluder devices (TEDs) in lieu of boosting hatchling survival through a headstarting program. In a similar vein, our sensitivity analysis results have important implications for predictions around the relative efficacy of various population management scenarios to be evaluated in this report.

It should be noted here that specific elasticity values emerging from this type of analysis are in part a function of the parameter values that make up the baseline model. Therefore, different modeling studies may come up with different elasticity values. Additionally, slight differences in model structure will also influence the results of an elasticity analysis (e.g., [7]). Nevertheless, previous studies showing higher sensitivity to survival estimates are in line with the results reported here, pointing toward the general robust nature of the methods used in this study.

**Supporting References**

1. Miller PS, Lacy RC (2005) *Vortex*: A stochastic simulation of the extinction process. Version 9.5 User’s Manual. Apple Valley, MN: IUCN-SSC Conservation Breeding Specialist Group.
2. Castillo D, Clarke AL (2003) Trap-Neuter-Release methods ineffective in controlling domestic cat “colonies” on public lands. Nat Areas Jour 23: 247-253.
3. Levy JK, Gale DW, Gale LA (2008) Evaluation of the effect of a long-term trap-neuter-return and adoption program on a free-roaming cat population. J Amer Vet Med Assoc 222: 42-46.
4. Centonze LA, Levy (JK) (2002) Characteristics of free-roaming cats and their caretakers. J Amer Vet Med Assoc 220: 1627-1633.
5. Jöchle W, Jöchle M (1993) Reproduction in a feral cat population and its control with a prolactin inhibitor, cabergoline. J. Repro. Fert. 47(Suppl): 419-424.
6. Nutter FB (2005) Evaluation of a trap-neuter-return management program for feral cat colonies: Population dynamics, home ranges, and potentially zoonotic diseases. Dissertation, North Carolina State University.
7. Andersen MC, Martin BJ, Roemer GW (2004) Use of matrix population models to estimate the efficacy of euthanasia versus trap-neuter-return for management of free-roaming cats. J Amer Vet Med Assoc 225: 1871-1876.
8. Foley P, Foley JE, Levy JK, Paik T (2005) Analysis of the impact of trap-neuter-return programs on populations of feral cats. J Amer Vet Med Assoc 227: 1775-1781.
9. Schmidt PM, Swannack TM, Lopez RR, Slater MR (2009) Evaluation of euthanasia and trap-neuter-return (TNR) programs in managing free-roaming cat populations. Wild Res 36: 117-125.
10. Warner RE (1985) Demography and movements of free-ranging domestic cats in rural Illinois. J. Wild. Manage. 49: 340-346.
11. Turner DC, Bateson P (2000) The domestic cat: The biology of its behaviour. Cambridge, England: Cambridge University Press. 244 p.
12. Levy JK, Friary JA, Miller LA, Tucker SJ, Fagerstone KA (2011) Long-term fertility control in female cats with GonaCon, a GnRH immunocontraceptive. Theriogenology 76: 1517-1525.
13. Caswell H (2001) Matrix population models. Sunderland, MA: Sinauer.
14. Crowder LB, Crouse DT, Heppell SS, Martin TH (1994) Predicting the impact of turtle excluder devices on loggerhead sea turtle populations. Ecol Appl 4: 437-445.
15. Akçakaya HR, Root W (2075) *RAMAS Metapop*: Viability Analysis for Stage-Structured Metapopulations (version 5). Setauket, NY: Applied Biomathematics.
